# Designing of the Electromechanical Drive for Automated Hot Plate Welder Using Load Optimization with Genetic Algorithm

**DOI:** 10.3390/ma15051787

**Published:** 2022-02-27

**Authors:** Krzysztof Wałęsa, Krzysztof Talaśka, Dominik Wilczyński

**Affiliations:** Institute of Machine Design, Poznan University of Technology, 61-138 Poznań, Poland; krzysztof.talaska@put.poznan.pl (K.T.); dominik.wilczynski@put.poznan.pl (D.W.)

**Keywords:** hot plate welding, plasticization, automated device, butt welding, drive belts, thermo-weldable belts, electromechanical drive system, optimization, genetic algorithm, design of the drive system, kinematic structure

## Abstract

Drive and conveyor belts are widely used in the mining and processing industry. One of the types often used is the belt with a cross-section and a diameter of several millimeters, made of weldable thermoplastic elastomer. Their production process requires the joining of the ends to obtain a closed loop. This operation is often performed by butt welding using the hot plate method. Taking into account the industrial requirements, the authors made an effort to design the automated welding machine for this type of belt. The work that had been conducted was finished with the implementation of the device for serial belt production. One of the stages of the design process of the welding machine consisted of developing a solution for the electromechanical drive system. The paper presents a design and the selection of the key components of the drive system, in particular, the electrical executive elements. Firstly, on the basis of the functional requirements of individual working mechanisms, the kinematic structure of the drives was developed, and the influence of the workload on power consumption was described. Then, using known technological parameters, experimental research of the plasticization operation was performed. On the basis of the results obtained, a mathematical model of the correlation between the plasticization force and technological parameters was derived. Using the derived model, the optimization of the technological parameters was made by using a genetic algorithm. The work led to the choice of an effective electric motor, which is the main component of the designed drive system.

## 1. Introduction

### 1.1. The Source of the Problem

Drive belts with a circular cross-section and a diameter of several millimeters are commonly used in industrial machinery drives and transport systems [1], similar to flat composite belts, which are often perforated [2]. In many cases, they are made from thermoplastic elastomers, in particular, polyurethane and polyester [3]. Their production process is two-step. In the first step, a belt with several hundred meters of length is produced by continuous extrusion. This stage ends with the belt being wound onto the spool, which facilitates transport and storage [4]. The final stage of the belt production process consists of cutting the belt to an appropriate length and making a permanent connection between its ends. This results in a drive belt with a specified circumference [5].

In many cases, the belt ends are connected by butt welding using the hot plate method. This solution is a cheap and easy way to join thermoplastic materials [6]. This process is usually conducted manually using simple dedicated tools. Unfortunately, this action does not provide satisfactory repeatability of their dimensions or high quality of welds. This is due to the manual application of the force needed to plasticize and connect the ends of the belt, which does not ensure the repeatability of the conditions of this process. In response to industry demand for the automation of hot plate butt welding of drive belts, the authors have proposed a solution for the belt welding, in the form of an automated welder.

During the design process, one of the most important issues is the design of the drive system. To do this properly, the following steps should be taken:

- Specifying the workload which acts on the executive mechanisms;
- Analysis of the structure of the drive unit to obtain a mathematical model of the connections between executive mechanisms and drive systems, considering entire loads and forces. To do this in a proper way, there is a necessity to perform the analysis of the load state of the mechanisms, using basic principles of mechanics, including the strength of the materials for particular parts;
- Estimation of the power consumption based on the workload of the drive system;
- Possible optimization of the power transmission system to obtain effective work parameters with low energy consumption.

### 1.2. Current State of Knowledge

A lot of researchers have conducted research on different aspects of the hot plate welding process. Watson [7] derived the correlation between the strength of the joint and the technological parameters during welding polypropylene, polystyrene, and polyphenylene oxide. He concluded that the heating time has a significant positive influence on the strength of the joint. Furthermore, he proved that the heating pressure and displacement, as well as pressure force, affect the strength ambiguously; additionally, this effect depends on the welded material. Gehde [8] worked on different joint defects and the correlation between their occurrence and welding parameters. His case study constituted beams made of polypropylene. He proved that the bond failure mechanism was dependent on the type of material and also on the welding parameters. According to his conclusions, increasing the joining pressure gives higher strength to the joint but could lead to the generation of a crack in the cross-section. Stokes [9,10,11] conducted a lot of research connected with the welding of plastics. His main area of interest was connected with correlations between the relative strength of the joint and the welding parameters. He did research on i.a. polycarbonate, where he obtained a results connected with the strength of the joint and failure deformation depending on the temperature of the hot plate, the heating and the sealing time, and preparation of the specimen [9]. He also obtains similar results for polycarbonate, polyetherimide, and poly(butylene terephthalate) [10]. Stokes [11] also obtained results connected with weld strength and failure strain for many standard polymer materials (e.g., acrylonitrile–butadiene–styrene, polyamide, polyethylenimine, etc.). His empirical studies were carried out with an interesting method of welding: pressure controlled with displacement restriction. His results are a type of database of welding parameters for rigid thermoplastic materials [10,11]. Potente [12,13,14,15,16] also researched the hot plate welding process, but his work mainly concerned mathematical modeling and process optimization, with regards to thermodynamic and rheological models. In [12] he showed the derivation of the mathematical model for one-dimensional hot plate welding. The obtained model was also verified experimentally. He proved that the complex nature of the thermal interaction between the hot plate, welded material, and the environment is present during hot plate welding. This point of view can be projected onto the device design process of the drive system of the device. Potente [13] also carried out research on using the different geometry of contact surfaces for the welding. Studying the conclusion of this work, gives us information about the lack of positive effects with regards to obtaining an enlarged surface area prior to plasticization on the final strength of the joint. In a similar way, as during the welding model derivation [12], Potente [14] also worked on simplifying this model using scaling thermodynamic and rheological laws. As a result of these works, he obtained a model for rigid materials which has non-dimensional coefficients. Potente [15] also tried to optimize the hot plate welding process using computer-aided design for self-optimizing heated tool welders. He also worked on high-speed welding for a designed machine, which was performed in cooperation with Schnieders [16]. In all of his works, the general principle of the welding operation is based on a pressure-controlled mechanism, without displacement control. Nonhof and Cocard [17,18] also tried to optimize the welding process for this type of control. Poopat [19] conducted research related to hot plate welding with an analysis of contact and contactless ways to carry out this technological operation. These articles can be treated as a sort of database for High-Density Polyethylene welding using the pressure-controlled method. Riahi [20] conducted research on plasticization polyethylene using analytical and numerical methods. He tried to find a correlation between the strength of the joint and the welding time or hot plate temperature. Oliveira [21] examined plasticization of monolithic and glass-reinforced polypropylene. The main objective of his work was the estimation of the size and structure of the flash with regards to the process parameters.

Several researchers also conducted work on the plasticization operation, which is one of the most important actions during the hot plate welding process. Wood [22] attempted to derive the model for this operation by using flow continuity equations and a classical thermodynamic approach. Yoo [23] obtained the rigid pipe plasticization model using the finite element method that includes Galerkin solutions. Ezekoye and Nieh [24,25] obtained a simplified model of plasticization for the purpose of predicting the weld strength. They used the theory of polymer chain diffusion and thermodynamic similarity numbers. Lee [26] obtained the correlation between process parameters and the degree of plasticity, using differential scanning calorimetry or thermogravimetric analysis.

Analysis of the current state of knowledge, concerning the technological process of hot plate welding indicates that:

- The research work which has been undertaken so far, is mainly connected with the butt welding of thermoplastics with relatively high rigidity at the standard environment temperature (polyamide, polycarbonate, polyethylene, and polypropylene);
- Analyzed cases of butt welding mostly concern elements with a complex cross-sectional shape and as a result, their susceptibility to buckling is low.

Therefore, the butt welding process is often described as a set of compression operations under variable temperature conditions. These phenomena occur in two stages of the process: during plasticization and the pressing of the surfaces together. Due to this fact, most researchers try to describe the process by analyzing the force (or pressure) of the material pressing. This approach is common, regardless of the deviations in practical implementation, which consist, for example, of the use of displacement limiters during plasticization.

In the authors’ opinion, the presented approaches for plastic hot plate welding are not suitable for the consideration drive belts. This is due to the specific properties of the welded items, which have simple and small cross-sections and low rigidity. In this case, the susceptibility to buckling is high. This phenomenon can cause unpredictable and undesirable effects during welding operations. As a consequence, the authors believe that the information presented in the literature, concerning the description of this technological process, is insufficient.

The connection between research and the device design process is crucial. Analysis of the state of knowledge about the design of automated technological devices, where engineering activity is related to research work, gives us interesting results. Wojtkowiak [27,28] analyzed the perforation of transport belts with a flat cross-section for the purpose of designing the automatic device. His achievements are related to the design of the automated perforating device and the optimization of the geometrical features of the working tool. The research carried out made it possible to obtain effective technological parameters that allow for a reduction of energy consumption. As we can see from this example, there is a correlation between the optimization of technological parameters and the features of the particular mechanisms of the designed device. The same situation can be observed in the case of the work of Górecki [29], who worked to optimize the dry ice compaction process, using the piston–die technique. Experimental research connected with mathematical modeling gave him the possibility to optimize the technological parameters of compaction, which ensures the lowest energy consumption possible. These works provide an opportunity for observing the connection between design and research.

Designing welding machines is also a popular topic with many references. Bahrami-Samani [30] conducted research on the welding robot design process. The main area of interest presented in his work is related to modeling the movement of the gripper assembly that is attached to the welding robot. Li [31] worked on the design of automatic welding machines and focused mainly on the configuration of the control system and the handling algorithm. The works of these two researchers have one common feature: they mainly focus on metal welding, so the design guidelines that are present in their works may not be useful for designing the automated welder for thermoplastic drive belts.

Many researchers have carried out work related to the design of devices for welding plastics. Unfortunately, devices that use the simple hot plate welding method are not very popular in the literature. Researchers more often describe the design guidelines for more sophisticated units, such as:

- Linear vibration welders [32];
- Spin welders [33];
- Devices that use a mixture of the pressure method and the frictional one, which is called friction stir welding [34];
- Devices for hot plate welding, but much more complicated, for example, with the use of infrared radiation [35].

Information from the literature about the design of the devices for different technological operations [27,28,29,30,31,32,33,34,35], especially for the aspect of plastics joining [32,33,34,35], is very helpful but does not provide satisfactory solutions for the butt welding of drive belts.

Genetic algorithms are a stochastic search technique, a little different from the usual mathematical programming. The search for solutions using this method is based on the mechanisms of natural selection, genetics, and Darwinian survival of the fittest [36,37]. This method is often used to assist during calculations, especially in optimization, and can be used during the machine design process. In the literature, there are many examples of considering genetic algorithms during the design process. They are often used for the optimization of electrical components, especially for the calculation of the necessary parameters of low-pass filters [38] or PID controllers [39].

Using genetic algorithms also makes it possible to design optimal mechanical constructions. They are often used to optimize the geometry of mechanical components, especially considering the strength of the materials. The most characteristic purpose is to optimize beams [40], frame structures [41], and some specific construction elements, for example, drive shafts [42].

When it comes to the utilitarian aspects of genetic algorithms for optimizing the drive of industrial devices and vehicles, the most common approach is related to the electrical aspects of drive systems instead of mechanical ones. They can be used for the optimization of the working parameters of servo drives [43], standard induction drives [44], or permanent magnet synchronous motors [45]. Genetic algorithms are also used to optimize the drive system as a whole, basically implemented in vehicle control systems [46] and other devices [47]. Adaptive techniques of calculations (which can be seen as a base of genetic algorithms) are often used for industrial purposes, especially in the motion control systems of different devices. There are known cases of the application of predictive [48] or completely adaptive [49] algorithms for controlling machines and industrial equipment. These solutions are distinguished by the ability to adapt the operation of the propulsion system to the currently encountered obstacles.

Referring to the simple hot plate welding process, in the authors’ opinion, there is a lack of knowledge on the literature concerning the following aspects:

- Building the mathematical model based on the technological parameters of the hot plate welding process using, for example, experimental data;
- Calculation of the gained model using the genetic algorithm to obtain the effective technological parameters for the purpose of estimating the minimal workload of the drive system;
- Consequently, designing the electromechanical drive system of the automated hot plate welder.

The works presented in the following paragraphs, in the authors’ opinion, make it possible to fill this area. In addition, the presented results will allow us to point out the connection between the hot plate welding operation itself, the standard design process, and the use of advanced mathematical methods such as genetic algorithms for obtaining the optimal solution of the main drive system.

## 2. The Solution of the Automated Hot Plate Welding Process

### 2.1. The Essence of Making the Butt Joints of the Round Belt

**Figure 1:**
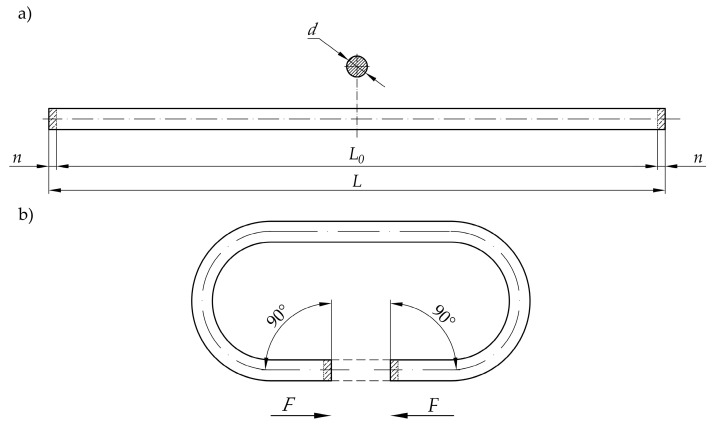
Preparation of the belt before the hot plate welding operation: (**a**) cutting to the proper length and (**b**) manipulation of the ends of the belt to obtain their proper orientation.

In general, preparing the drive belt with the butt weld that is made by the hot plate method, consists of two main stages (Figure 1):

- Belt preparation that consists of unwinding the belt from the spool, measurement, cutting to the proper length, and placing the cut belt in the appropriate mechanism, which makes it possible to conduct proper manipulation that allows joining execution;
- Conducting the proper welding operation.

During the preparation of the belt for the welding operation, it is necessary first to unwind the belt from the spool on which the belt is transported. The belt is then cut to a starting length *L*, where an additional material with the length *n* is included for both sides. This allowance will be melted during the welding operation. Due to this, the final length of the belt will be *L*_0_, making it possible to obtain a strand with the desired circumference (Figure 1a).

The following operations during belt preparation consist of placing the belt in a gripping system in order to perform the manipulations of the end of the belt in an automated way. This action should be taken so that the flat surfaces at the end of the belt should be parallel and coaxial (Figure 1b). The manipulation system, which is used in the automated welding device, should allow the application of the axial force *F* to perform further operations during the welding process.

### 2.2. The Hot Plate Welding Process

**Figure 2:**
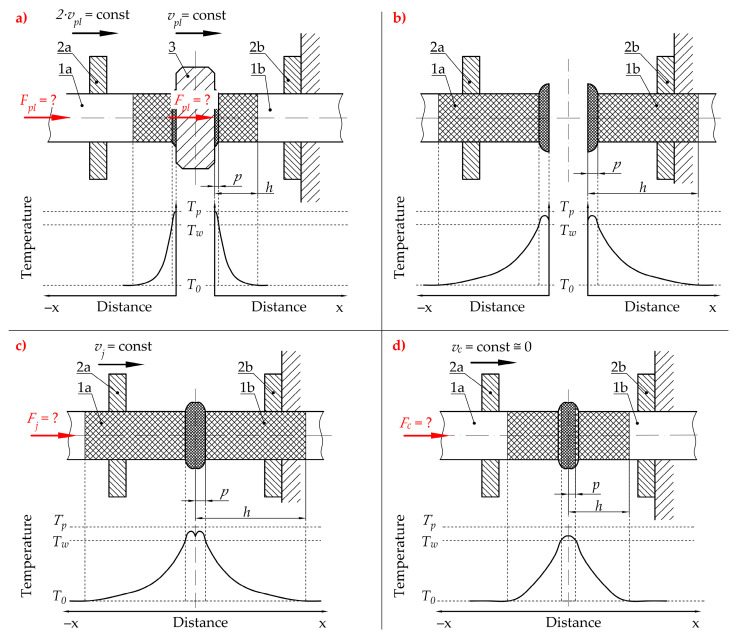
Diagram of the hot plate welding process of the drive belt divided into 4 phases: (**a**) plasticization, (**b**) removing the heating device, (**c**) joining (pressing), (**d**) cooling of the joint; 1a—movable end of the belt, 1b—fixed end of the belt, 2a—movable belt holder, 2b—fixed belt holder, 3—hot plate; *v_pl_*—plasticization velocity, *v_j_*—velocity of pressure during joining, *v_c_*—velocity of pressure during cooling, *F_pl_*—pressure force during belt plasticization, *F_j_*—pressure force when connecting the belt ends, *F_c_*—pressure force during cooling of the connection, *T_p_*—temperature of the hot plate, *T_w_*—welding temperature, *T*_0_—environment temperature, *p*—belt plasticization distance, *h*—distance heated to a temperature higher than environment temperature.

Referring to the unsatisfactory state of knowledge available in the literature, and considering the industrial requirements, the authors decided to introduce their own method for conducting the welding process of the belts for their serial production. Unlike typical methods, which are described in the literature, where the welding process is divided into five phases [50,51], the approach introduced by the authors considers the division of the entire process into four main phases (Figure 2):

- The plasticization of the belt (Figure 2a), is when the hot plate (3) is pressed to the fixed end of the belt (1b), which is mounted in the fixed belt holder (2b). This is a consequence of a movement of the plate (3) with controlled velocity *v_pl_*. Simultaneously, the second end of the belt (1a), which is mounted in the movable belt holder (2a), is pressed to the hot plate (3) from the other side, with controlled velocity 2·*v_pl_*. Finally, the relative velocity between the hot plate (3) and fixed end (1b), as well as between the movable end (1a) and hot plate (3), is equal to *v_pl_*. The most important feature of this assumption, according to the authors’ intention, consists of simultaneous displacement of the movable end of the belts (1a) and the hot plate (3), which gives the effect of uniform plasticization of both ends of the belt (1a and 1b). Additionally, the plasticization force value *F_pl_* is variable and not regulated because of displacement control, which is easier to implement in industrial conditions. The main objective of this operation is to elevate the temperature of the ends of the belt to a value which is almost equal to the plate temperature *T_p_*, which is higher than the welding temperature *T_w_* (which is given as a material property). This temperature correlation will ensure that the temperature *T_w_* is obtained during the proper joining operation (considering the energy losses due to convection and radiation during the next phases [52]);
- Removing the hot plate (Figure 2b) is when the heating device (3 in Figure 2a) is retracted from the area between the ends of the belt (1a and 1b). At this stage, the heating process in finished. Unfortunately, the ends of the belt (1a and 1b) are cooled by convection with the environment and radiation. For this reason, this stage should be as short as possible to avoid situations when the flat surface of the ends of the belt (1a and 1b) will be cooled down to a temperature which is lower than the welding temperature *T_w_*;
- The pressing phase (Figure 2c) is when the movable end of the belt (1a) is pressed against the fixed one (1b) with velocity *v_j_*. This activity is crucial for the overall process. The thermal activation of the belt surface (flat surface of the ends of the belt 1a and 1b) and pressing force at this stage leads to contact between them; therefore, this is where the interactions between the ends (1a and 1b) of the belt begin. They consist of mechanical interactions between polymer macromolecules which lead to their splicing. This phenomenon facilitates the start of the chemical reactions between them [53], which is an action that allows for obtaining a connection. In this phase, only the velocity *v_j_* is known and regulated which facilitates the precise control of the operation;
- The cooling phase (Figure 2d) is when the movement of the movable end of the belt (1a) is stopped and compression velocity *v_c_* is approximately equal to 0. Despite the fact that there is no pressure to compress the joint, the ends of the belt (1a and 1b) can be pressed together by residual force *F_c_* which might remain after the compression process (Figure 2c). During this phase (Figure 2d), the joint cools down as a result of heat exchange with the environment (convection and radiation). In the ideal situation, the joint should remain in this state for a period of time, allowing its temperature to be lowered to *T*_0_. The main assumptions of this phase are connected with equalization of the temperature between the belt ends and the environment and finalization of the chemical reactions and physical interactions between macromolecules.

The introduced hot plate welding cycle, in terms of industrial utilization and customer requirements, gives the following positive functional features:

- Precise control of the final length of the belt *(L*_0_ in Figure 1), omitting the standard problems during controlling small-value forces, due to displacement regulation;
- Simplifying the control system framework and lowering the complexity of the mechanical design due to using just one drive unit for performing the main technological movements.

### 2.3. The Conception of Automated Hot Plate Welder

**Figure 3:**
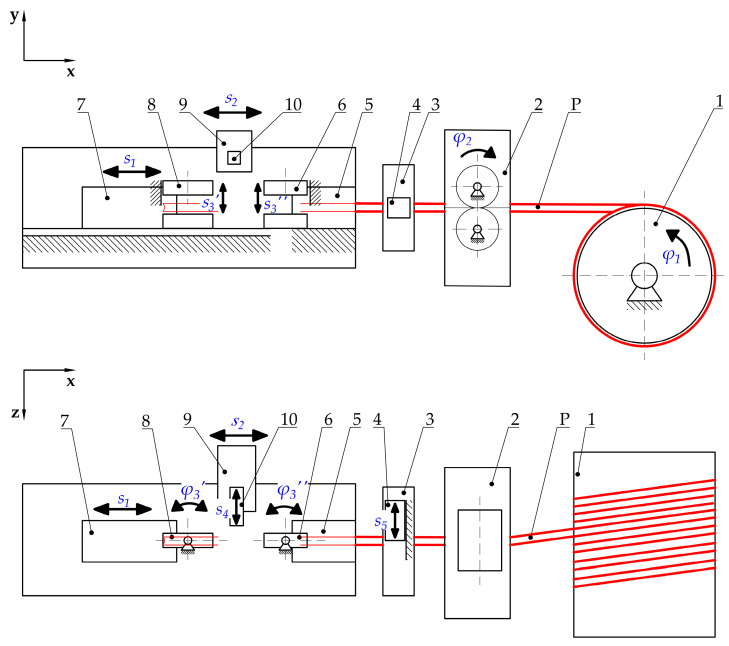
The concept of the automatic hot plate welder: P—belt, 1—spool with wound belt, 2—roller feeder, 3—cutting unit, 4—knife cutting system, 5—fixed belt holder, 6—jaw of fixed belt holder, 7—movable belt holder, 8—jaw of movable belt holder, 9—heating unit, 10—hot plate; *s*_1_*…s*_5_—linear displacement, *φ*_1_*…φ*_3_—angular displacement.

To ensure the possibility of implementing the proposed belt welding cycle, the authors have developed the concept of an automatic welding device (Figure 3).

With this device, the belt (P) is taken from a spool (1), which can rotate freely. The roller feeder (2) subsequently transports the belt from the spool (1) to the cutting unit mounted on its housing (3). This mechanism (3) is equipped with a knife cutting system (4), which is responsible for cutting the belt perpendicularly to its central axis. Therefore, the roller feeder and cutting assembly (2 and 3) have the ability to:

- Dose the belt (P);
- Make a measurement of the length of the dosed belt;
- Cut the belt in a suitable place.

The cut end of the belt (P) is later fed through the fixed belt holder (5) to the movable belt holder (7). After that, its rotary jaw (8) clamps by moving the upper part down in the direction *s*_3_*′*, resulting in the end of the belt being grabbed (P). Then, the movable belt holder (7) with the clamped belt (P) moves to the left according to the displacement *s*_1_, while dosing the belt (P) by the roller feeder (2) simultaneously. After giving the appropriate length of the belt (P), the movable belt holder (7) stops similarly to the roller feeder (2). After that, the jaw (6) of the fixed belt holder (5) clamps the second end of the belt (P), moving downward according to the direction *s*_3_*″.* These actions result in the location of both ends of the belt being specified.

In the next sequence, the knife (4) cuts the belt (P) by performing the movement according to the displacement *s*_5_. Because of this, the first stage of belt preparation is performed, the dosing of the belt, and the specification of its proper length (Figure 1a).

Manipulation of the ends of the belts to obtain their proper orientation (Figure 1b) consists of performing their rotation by 180° using the possibility of the controlled rotation of the jaws (6 and 8) with a clamped belt (P), according to the directions *φ*_3_*′* and *φ*_3_*″* (Figure 3). After that, the movable belt holder (7) moves in the direction *s*_1_ towards the fixed belt holder (5), resulting in the ends of the belt (P) approaching each other. When the distance between them becomes small enough (considering the thickness of the hot plate (10) enlarged by a small allowance), the movable belt holder (7) stops. This action results in the completion of the belt preparation to make a connection.

The joining operation starts with the heating of the belt on the surface of the hot plate (Figure 2a), resulting in the plasticization process. To do this correctly, the hot plate (10), which is mounted on the heating unit (9) is heated until the *T_p_* temperature is reached (Figure 2a and Figure 3). The hot plate (10) is then linearly moved according to direction *s*_4_*,* until it is placed between the ends of the belt (P) (Figure 3).

Moving of the hot plate (10) is a moment when the plasticization operation starts (Figure 2a). The movable belt holder (7) with the end of the belt (P) clamped starts to move toward the fixed belt holder (5) according to the direction *s*_1_ (Figure 3). This action results in the end of the belt (P) held in the movable belt holder (7) approaching the hot plate (10). At the same time, to obtain the phenomenon of reducing the distance between the end of the belt (P), which is attached to a fixed belt holder (5), and the hot plate (10), a specific configuration of the drive system is used. The drive units of the movable belt holder (7) and of the heating unit (9) are connected in such a way that their simultaneous displacements can be described by the following formula (Figure 3):

(1) $$s_{1}=2\cdot s_{2}.$$

Accordingly, the heating unit (9) moves in the same direction as the movable belt holder (7) but with a displacement (or velocity) that is twice lower. Due to this, during the plasticization operation, the end of the belt (P) that is mounted on the movable belt holder (7) is pressed to the hot plate (10) identically to a hot plate (10) which is pressed to the end of the belt (P) that is mounted on the fixed belt holder (5). This is a result of maintaining the same relative velocity between these elements (7 and 10, 5 and 10).

In the next step, after obtaining the required plasticization distance, the hot plate is removed from the heating area (Figure 2b) by moving in the direction *s*_4_ (Figure 3). This operation makes it possible to start the next step, the pressing (joining) operation (Figure 2c). The proper flow of this operation consists of moving the movable belt holder (7) towards the fixed belt holder (5) according to the direction *s*_1_ (Figure 3). After obtaining the desired displacement *s*_1_, the movable belt holder (7) is stopped, which allows us to start the cooling phase (Figure 2d).

The presented conception of the hot plate welder makes it possible to prepare the belt and make the proper connection in an automated way. The device allows for making butt joints of thermo-weldable belts with the round cross-section and specified two dimensions: diameter and circumference length.

### 2.4. Industrial Implement of the Automated Welding Machine

**Figure 4:**
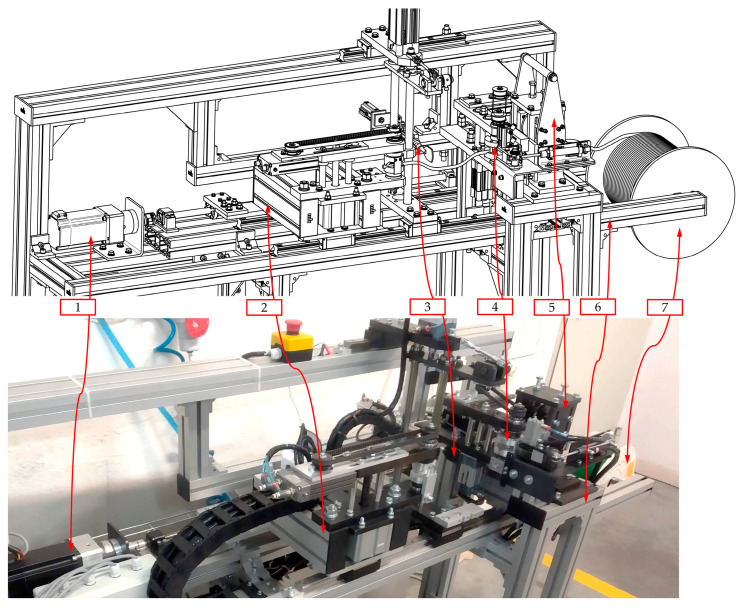
The prototype of the automatic welding machine: 1—the main drive unit of the device, 2—movable belt holder assembly, 3—heating unit, 4—fixed belt holder assembly, 5—roller feeder assembly with cutting unit, 6—frame, 7—spool with the belt wounded.

**Figure 5:**
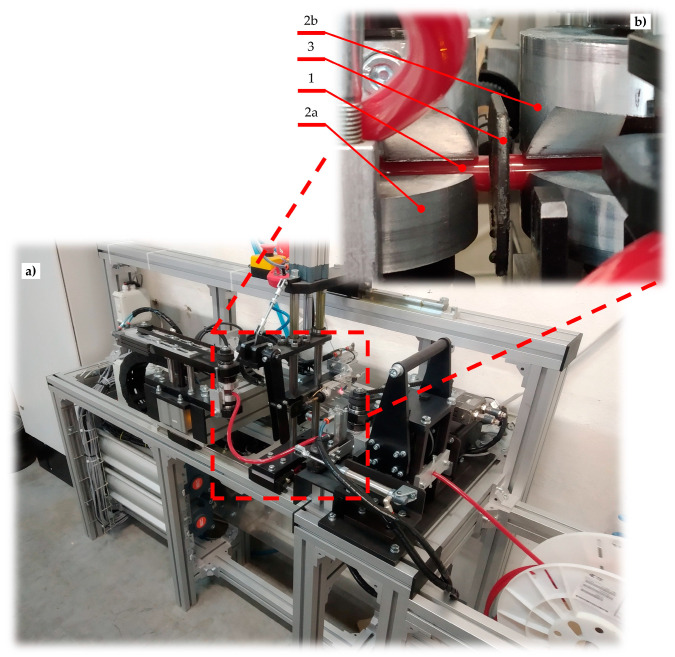
Automatic welding machine during butt welding of polyamide drive belt: (**a**) overview during belt dosing, (**b**) focus on the hot plate unit during the plasticization process; 1—belt, 2a—grips of the movable belt holder, 2b—grips of the fixed belt holder, 3—hot plate.

The proposed design of the automated hot plate welder was manufactured and introduced into the industrial production of drive belts. The final prototype of the device is presented in Figure 4 from a general view and in Figure 5 during dosing (Figure 5a) and plasticization (Figure 5b) of the polyester belt.

The introduced device has a modular construction where particular units can cooperate with the others (Figure 4) and are capable of performing the specified functions. The set of the roller feeder and cutting unit (5) is responsible for removing the belt from the spool (1), dosing it, and cutting to the proper length. These units make it possible to obtain the segment of the belt with length *L* (Figure 1), that will allow obtaining the final belts with the desired circumference after welding.

During belt preparation, the roller feeder (5) cooperates with the movable belt holder (2) and the fixed belt holder (4) to (Figure 4):

- Ensure the proper leading of the belt through all of the mechanical parts;
- Protect against the tangling of the belt by obtaining the initial tension of the strand;
- Make the required manipulation of the ends of the belt in order to achieve proper orientation.

The welding operation is possible due to the cooperation between the belt holders (2 and the 4) and heating unit (3). The set of the movable belt holder (2) and the heating unit (3) is driven by a common drive unit (1), which ensures proper correlation between the displacements of these component modules (Equation (1)). Due to this, the simultaneous pressing effect is gained during the plasticization operation. This solution also makes it possible to simplify the construction of the device.

All modules are placed in the common modular frame (6), which is made of shaped aluminum profiles. Profiles are connected by specialized joining elements. This configuration provides the possibility for the reconfiguration or expansion of the device.

The grips of the movable belt holder (2a) and the fixed belt holder (2b) have specialized shapes to obtain the proper leading of the belt (1) (Figure 5). This construction makes it possible to obtain a coaxial relationship between the ends of the belt during both the plasticization operation and the pressing phase (Figure 1). At the same time, the perpendicularity between the surface of the hot plate (3) and the axis of the ends of the belts (1) is preserved (Figure 5).

The presented device allows the belt to be welded with a minimal diameter *d* = 3 mm and minimal length *L* = 180 mm (Figure 1). There are no restrictions when it comes to the maximum length of the belt; however, the maximum diameter is limited by the shape of the grips of the belt holder (2a and 2b), but the target value is *d* = 18 mm (Figure 5).

## 3. Design of the Main Drive

### 3.1. Structure of the Main Drive

**Figure 6:**
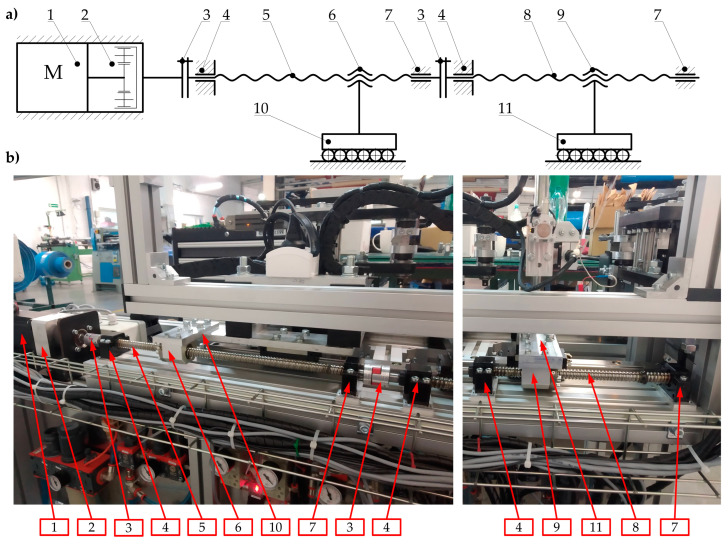
The main drive system of the automatic welding machine: (**a**) kinematic structure of the drive mechanism, (**b**) practical implementation of designed drive unit in manufactured machine;1BLDC electric motor, 2—planetary gear, 3—jaw coupling with flexible insert, 4—angular bearing unit (fixing), 5—ball screw with pitch *p*_1_, 6—ball nut with pitch *p*_1_, 7—radial bearing unit (floating), 8—ball screw with pitch *p*_2_, 9—ball nut with pitch *p*_2_, 10—movable belt holder assembly, 11—heating unit assembly.

The kinematic structure with the practical implementation of the main drive of the automated hot plate welder is presented in Figure 6.

In this solution, the BLDC electric motor (1), in cooperation with the built-in planetary gear (2), through the jaw coupling with elastomer insert (3), drives the ball screw mechanism of the movable belt holder (10). This mechanism consists of a screw (5) and ball nut (6). The screw is supported by two ball bearings. The first bearing (4) is angular and can carry radial and axial loads. This support can be treated as a fixed one. The second bearing (7) can carry only radial loads, so it can be treated as a floating one.

The last journal of the first screw (5) is connected to the second jaw coupling (3), which drives the ball screw mechanism of the heating unit (11). This mechanism consists of the second screw (8) and the ball nut (9). Similar to the first screw (5), the second one (8) is supported by two bearings (4 and 7) in the same configuration.

The most important feature of this drive system is the fact that the first screw (5) (which cooperates with the movable belt holder (10)) has a pitch *p*_1_ and the second screw (8) (which cooperates with the heating unit (11)) has a different pitch—*p*_2_. The correlation between the pitch values for both screws has the following form:

(2) $$p_{1}=2\cdot p_{2}$$

Assuming the same rotational speed of these two screws (5 and 8), the proper correlation (Equation (1)) between displacements of the movable belt holder (10) and the heating unit (11) can be obtained.

**Figure 7:**
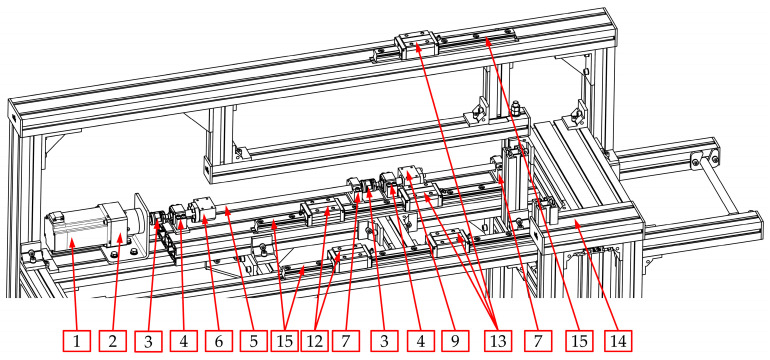
The main drive and guiding system of the automatic welding machine: 1—BLDC electric motor, 2—planetary gear, 3—jaw coupling with flexible insert, 4—angular bearing unit (fixing), 5—ball screw with pitch *p*_1_, 6—ball nut with pitch *p*_1_, 7—radial bearing unit (floating), 8—ball screw with pitch *p*_2_, 9—ball nut with pitch *p*_2_, 12—carriages of the guiding of the movable belt holder, 13—carriages of the guiding of the heating unit, 14—structural frame, 15—profiled rail.

One of the most important components of the drive system, in addition to the screw mechanism, are the linear guideways of the movable belt holder and the heating unit (Figure 7). Guideways are responsible for linear motion in one direction. Additionally, linear guideways make it possible to fix the remaining degrees of freedom, so they transfer all the forces that act in directions different from the direction of movement (*s*_1_ and *s*_2_ in Figure 3).

The linear guiding of the movable belt holder and the heating unit consists of cooperation between carriages (12 and 13) with profiled rails (15). The trolleys (12 and 13) are screwed into the structural elements of the movable belt holder—two carriages (12) and to the heating unit—three carriages (13). On the other hand, the rails are common to both units and are connected to the structural frame (14). The designed guidance is of the rolling type, so relatively small energy losses can be obtained due to friction (Figure 7).

### 3.2. The Load of the Main Drive

**Figure 8:**
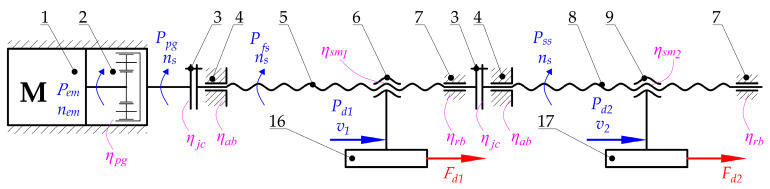
The kinematic structure of the main drive system of the automatic welding machine: 1—BLDC electric motor, 2—planetary gear, 3—jaw coupling with flexible insert, 4—angular bearing unit (fixing), 5—ball screw with pitch *p*_1_, 6—ball nut with pitch *p*_1_, 7—radial bearing unit (floating), 8—ball screw with pitch *p*_2_, 9—ball nut with pitch *p*_2_, 16—handle of the movable belt holder, 17—handle of the heating unit; *P_em_*—output power of the motor, *P_pg_*—output power of the planetary gear, *P_fs_*—power in the first ball screw, *P_ss_*—power in the second ball screw, *P_d_*_1_—power in the movable belt holder mechanism, *P_d_*_2_—power in heating unit mechanism, *n_em_*—rotational speed of the electric motor, *n_s_*—rotational speed of the screws, *v*_1_—linear velocity of the movable belt holder, *v*_2_—linear velocity of the heating unit, *F_d_*_1_—force which is needed to drive the movable belt holder, *F_d_*_2_—force which is needed to drive heating unit, *η_em_*—efficiency of planetary gear, *η_jc_*—efficiency of jaw coupling, *η_ab_*—efficiency of angular bearing, *η_rb_*—efficiency of radial bearing, *η_sm_*_1_—efficiency of first screw mechanism, *η_sm_*_2_—efficiency of second screw mechanism.

**Figure 9:**
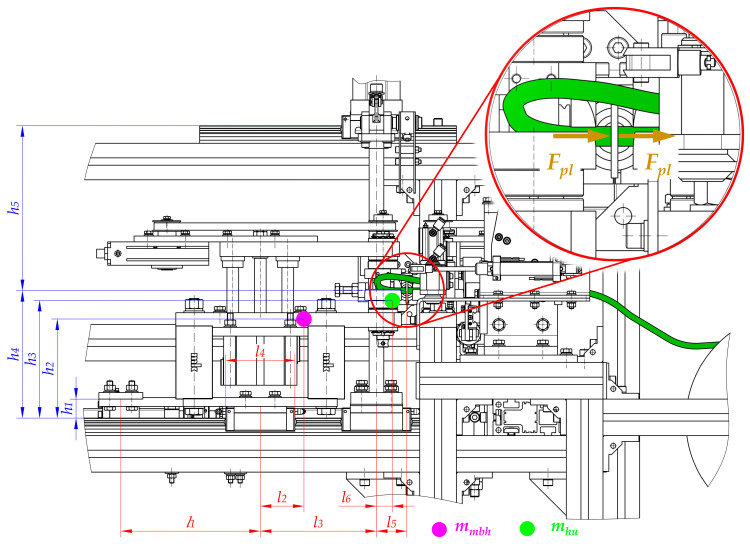
Horizontal view in a plane parallel to the linear guideways, of the automated hot plate welder during plasticization, with the particular dimensions which are necessary for the load state calculations: *m_mbh_*—center of mass of the movable belt holder, *m_hu_*—center of mass of the heating unit, *F_pl_*—plasticization force; *l*_1_*…l*_6_—particular values of length, *h*_1_*…h*_5_—particular values of height.

**Figure 10:**
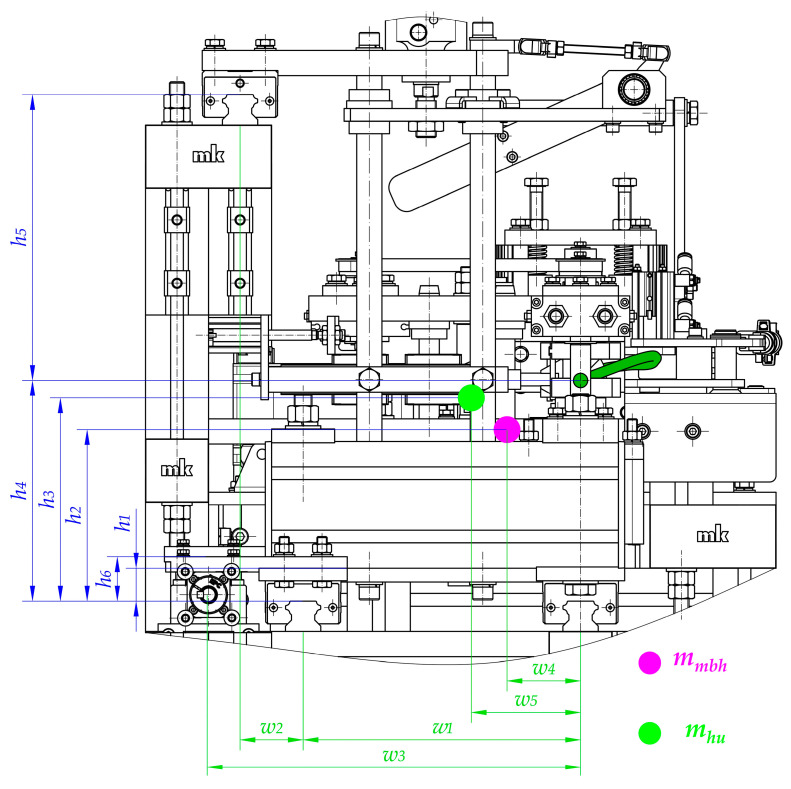
Vertical view in a plane that is perpendicular to the linear guideways, of the automated hot plate welder during plasticization, with the particular dimensions necessary for the load state calculations: *m_mbh_*—center of mass of the movable belt holder, *m_hu_*—center of mass of the heating unit, *w*_1_*…w*_5_—particular values of width, *h*_1_*…h*_6_—particular values of height.

**Figure 11:**
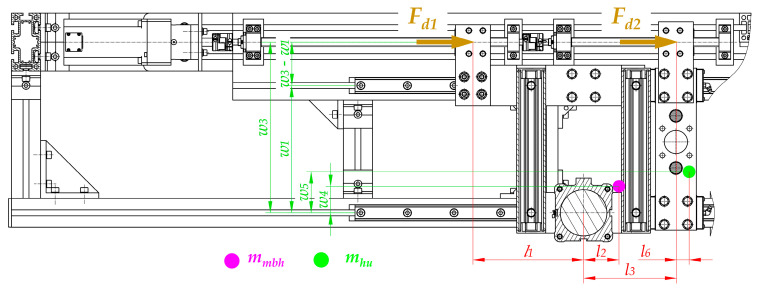
Horizontal section in a plane that is parallel to the linear guideways, of the automated hot plate welder during plasticization, with the particular dimensions necessary for the load state calculations: *m_mbh_*—center of mass of the movable belt holder, *m_hu_*—center of mass of the heating unit, *w*_1_*…w*_5_—particular values of width, *l*_1_*…l*_6_—particular values of length, *F_d_*_1_—force required to drive the movable belt holder, *F_d_*_2_—force necessary to drive the heating unit.

The correct selection of components of the welding device, in particular the electric motor, is crucial. To determine the required power of the designed propulsion system, the load should first be analyzed (Figure 8). Second, the special features of key components have to be taken. Consequently, the following issues should be considered:

- The values of the efficiency of particular components, especially planetary gear (2), couplings (3), angular and radial bearings (4 and 7), and screw mechanisms (5 and 6, 8 and 9) due to inevitable energy losses resulting from friction;
- Calculations that make it possible to transform the forces that are necessary to drive particular mechanisms (*F_d_*_1_ and *F_d_*_2_) for the required power of the motor (*P_em_*);
- Calculations that make it possible to transform technological forces (especially the plasticization force *F_pl_*) into forces that are necessary to drive particular mechanisms (*F_d_*_1_ and *F_d_*_2_). These types of calculations should be performed regarding particular distances between guiding elements and places where the technological forces act. In addition, the mass of the parts should be considered to obtain all of the force components (Figure 9, Figure 10 and Figure 11).

Particularly noteworthy is the fact that the calculations of the force that is necessary to drive particular mechanisms (*F_d_*_1_ and *F_d_*_2_) require the determination of the load state for each unit. A special function in the designed driving system is performed by linear guideways (12, 13, and 15 in Figure 7) because they carry all of the loads that act in this construction. Due to the fact that the geometry is complex and there is a three-dimensional load state, it is necessary to take into account the following features during calculations (Figure 9, Figure 10 and Figure 11):

- Distances between the carriages of the linear guideways (*h*_4_ + *h*_5_, *l*_3_, *w*_1_, *w*_2_);
- Size and length of the carriages (*l*_4_)—the same for all of guideways;
- Distances between the places where technological forces act and the carriages of linear guideways (*h*_4_, *h*_5_, *l*_5_, *l*_5_ + *l*_3_, *w*_1_, *w*_1_ + *w*_2_);
- Distances between the places where the driving force will be applied and the carriages of linear guideways (*h*_1_, *h*_6_, *l*_1_, *w*_3_, *w*_3_ − *w*_1_);
- Values of the masses of the movable belt holder *m_mbh_* and the heating unit *m_hu_*;
- Distances between the center of the mass of the movable belt holder and the carriages of linear guideways (*h*_2_, *l*_2_, *w*_4_);
- Distances between the center of the mass of the heating unit and the carriages of linear guideways (*h*_3_, *l*_6_, *w*_5_);
- Rolling friction coefficient between carriages and rails *μ*_1_.

Of course, detailed calculations can be performed after the estimation of the technological force that determines the load of the state of the construction. Calculations will be made for the most crucial operation, which is the plasticization of the belt.

## 4. Estimation of the Workload Value during the Plasticization Process

### 4.1. Tests of the Plasticization Process

**Figure 12:**
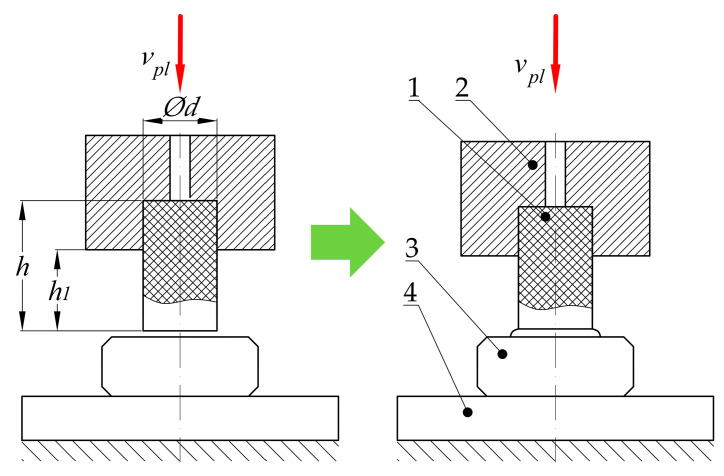
Diagram of the experiment of pressing the belt to the hot plate: 1—belt specimen, 2—specimen holder, 3—hot plate, 4—thermal insulation pad, *h*—specimen length, *h*_1_—specimen protrusion distance, *v_pl_*—test speed (plasticization velocity).

**Figure 13:**
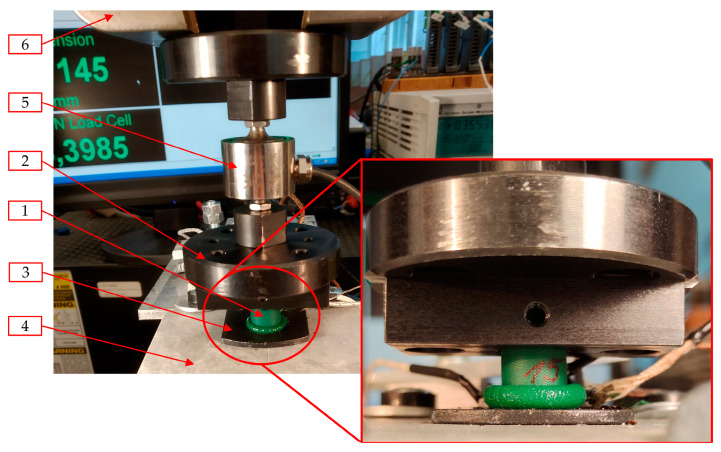
Test station during the belt plasticization process: 1—belt specimen, 2—specimen holder, 3—hot plate, 4—thermal insulation pad, 5—force sensor, 6—strength testing machine gripper.

To estimate the power consumption of the main drive system used in the automated welding machine, experimental research of the plasticization operation was conducted (Figure 12 and Figure 13). The experiment was carried out with respect to the welding of the belt made of thermoplastic polyurethane elastomer with the trade designation TPU C85A. For this material, the welding temperature of *T_w_* is usually between 250 and 310 °C [3,5,54,55].

The experiment consisted of pressing the cylindrical belt specimen (1), with the diameter *d* and height *h,* onto the hot plate (3), which was heated to the temperature *T_p_*. Specimen (1) was clamped in the specimen holder (2) in a way that the free part of it protruded from the holder (2) at distance *h*_1_. The heated plate (3) was placed on the thermal insulation pad (4) to avoid excessive energy losses and, consequently, unpredictable changes in temperature. The experiments were carried out on strength testing machine MTS Insight 50 kN, which was equipped with an additional force sensor HBM U1 with a measurement range equal to 1 kN (5). The sensor (5) was placed between the machine grips (6) and the specimen holder (2). Using an additional sensor with a smaller measuring range allowed one to decrease the percentage of measurement error.

The experiments were carried out for cylindrical specimens, which had been cut from belts of three different diameters *d*. For each type of specimen, the plasticization operation was performed for three different temperatures of the hot plate *T_p_*. For all of the diameters of specimen *d* and for all of the temperatures *T_p_*, the three different plasticization velocities *v_pl_* were used. Therefore, the 27 combinations of experimental parameters were considered. For each combination, five repetitions were made, which gives 135 experiments. As a consequence, there were the following variables during the test:

- Diameter of the belt *d*;
- Temperature of the hot plate *T_p_*;
- Plasticization velocity *v_pl_*.

The values of the parameters used are presented in Table 1. The test parameters were varied in a discrete way. Maximum displacement during the *s_MAX_* tests was set at 6 mm.

### 4.2. Results of the Experimental Research

**Figure 14:**
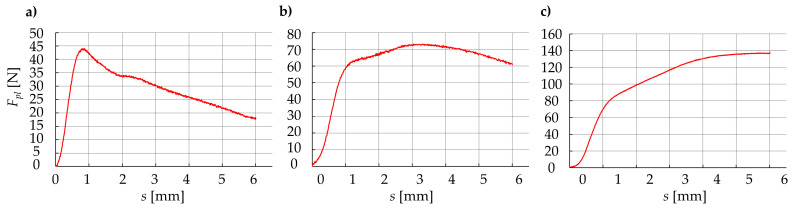
Exemplary results of the plasticization tests for the belt with diameter *d* = 12 mm, using the hot plate heated to *T_p_* = 285 °C: (**a**) plasticization velocity *v_pl_* = 6 mm/min, (**b**) plasticization velocity *v_pl_* = 8 mm/min, (**c**) plasticization velocity *v_pl_* = 10 mm/min.

The exemplary averaged results, for the belt with the diameter *d* = 12 mm, the hot plate temperature *T_p_* = 285 °C, and the different plasticization velocity *v_pl_*, are presented in Figure 14. The results obtained for all specimens (for the same parameters) were relatively repeatable. The standard deviations from the average values usually did not exceed 15%.

Considering the dependency between the plasticization force *F_pl_* and the displacement during plasticization *s* for particular groups of parameters, all the obtained results can be divided into three main groups:

- Value of the plasticization force *F_pl_* first increases almost linearly, then decreases non-linearly (Figure 14a);
- Value of the plasticization force *F_pl_* first increases almost linearly, then it is almost constant with little nonlinear deviation (Figure 14b);
- Value of the plasticization force *F_pl_* first increases almost linearly, then it also increases but in a nonlinear way (Figure 14c).

The existence of this phenomenon is independent of the diameter of the belt *d*, which means that it can be observed for all values of diameter. These types of differences in plasticization force characteristics *F_pl_*(*s*) can be noticed mostly during the comparison between different hot plate temperatures *T_p_* and plasticization velocity *v_pl_* for the same diameters *d*. Therefore, this phenomenon should be analyzed more carefully.

### 4.3. Discussion of the Results

**Figure 15:**
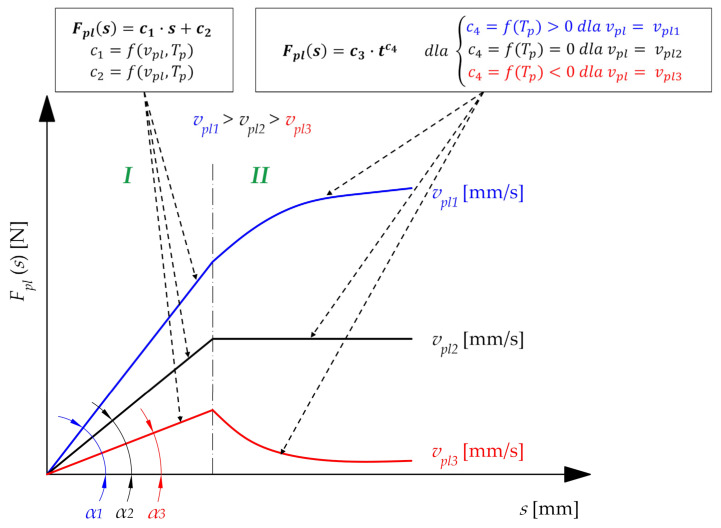
The analysis of the plasticization operation on the hot plate: *I*—steady-state plasticization, *II*—non-steady plasticization; *F_pl_*—plasticization force, *T_p_*—hot plate temperature, *s*—displacement, *v_pl_*_1_…*v_pl_*_3_—plasticization velocity, *c*_1_…*c*_4_—constant factors, *α_i_*—angle of inclination of the proportional section of plasticization correlation.

According to observations on the correlation between the plasticization force *F_pl_* and displacement *s* (Figure 14), there is the possibility of drawing a group of model characteristics, enabling the analysis of the collected data (Figure 15). Based on that, the plasticization characteristic *F_pl_*(*s*) can be divided into two main phases:

- Phase I, where the plasticization operation can be described as a steady state (almost linear, positive correlation between force *F_pl_* and displacement *s*);
- Phase II, when the plasticization operation can be described as transient (the correlation between the force *F_pl_* and the displacement *s* strongly depends on the current technological parameters).

Proportional increase of the plasticization force *F_pl_* in the first phase of the plasticization operation is due to the fact that the heating process of the belt is not instantaneous. Unlike assumptions for describing the heat transfer during this operation, which are often used and connected with modeling this phenomenon as a one-sided heating of a semi-infinite body (heating from its surface) [10,12,51,56], the heat exchange between the specimen and the hot plate during real plasticization is involved with certain thermal resistance [52,57,58]. In addition, not instantaneous heating is a result of a finite value of the thermal diffusion coefficient *a*, which implicate the effect of thermal inertia [59].

Assuming the fact, that the plasticization velocity *v_pl_* is constant and non-zero, in this phase there is compression of the partial unplasticized solid. Considering this phenomenon, a linear increase in the pressing force *F_pl_* is expected.

Based on observations of the results obtained, the directional coefficient of this straight line *c*_1_ (Figure 15) changes its value with the change in velocity *v_pl_*, diameter *d* and temperature *T_p_*_._ However, the following conclusions can be drawn:

- Assuming a constant diameter of the belt *d* and a constant temperature of the hot plate *T_p_*, there is a positive correlation between coefficient *c*_1_ and plasticization velocity *v_pl_*;
- Assuming a constant diameter of belt *d* and plasticization velocity *v_pl_*, there is a negative correlation between coefficient *c*_1_ and temperature of the hot plate *T_p_*;
- Assuming a constant plasticization velocity *v_pl_* and a constant temperature of the hot plate *T_p_*, there is a positive correlation between proportionality coefficient *c*_1_ and diameter of the belt *d*.

These observations make it possible to confirm the physical interpretation made earlier.

The observed changes in the plasticization characteristics *F_pl_*(*s*), during the second phase of the operation (Figure 15) depend on the constant and non-zero velocity *v_pl_,* diameter *d*, and temperature of the hot plate *T_p_*. This phenomenon is caused by limited heating velocity, which is a consequence of the finite value of a thermal diffusion coefficient *a*, which is also approximately independent of the plasticization velocity *v_pl_*.

During analysis, the influence of the plasticization velocity *v_pl_* to obtain characteristics *F_pl_*(*s*), three types of phenomena can be observed (Figure 15). The lower value of this parameter (*v_pl_*_3_) implicates a situation where the material is overheated. This is a result of small specimen velocity (*v_pl_*) relative to the heating velocity. These phenomena cause significant decrease in the value of the plasticization force *F_pl_*, as a result of the compression of the material subject to plastic flow. The observable effect of this type of plasticization is the melting and outflowing of the material from the working area. In industrial practice, this phenomenon leads to additional technological problems—outflowing molten polymer from the working area, can result in distorted joints or unnecessary pollution of the device. Therefore, it can be concluded that such plasticization is excessive, which leads to the unnecessary loss of energy and material. On the other hand, the higher value of the plasticization velocity (*v_pl_*_1_) leads to under-heating of the belt. This indicates a situation where the velocity of pressing the specimen onto the plate *v_pl_* is significantly higher than the velocity of heat transfer in the polymer structure. Consequently, material is not sufficiently plasticized, which indicates compression at a solid state. During the experiment, the observable effect of this situation is the increase in the plasticization force *F_pl_*, especially at the end of the phase II. In this case, the core of the material does not have a sufficient temperature or degree of plasticity to form a permanent welded connection. This situation is disadvantageous because the joint might be improperly connected, and the energy efficiency of the process decreases significantly. Finally, approximately intermediate plasticization velocity (*v_pl_*_2_) gives the effect of proper plasticization. For the plasticization characteristic *F_pl_*(*s*), there is a neutral correlation between the plasticization force *F_pl_* and displacement *s*, which indicates an almost constant course of the function. The physical interpretation of this phenomenon is that there is a balance between heat transfer and sample deformation. Such a course of the plasticization operation can be considered effective.

Considering the optimal design of the automated welding machine, the most interesting parameter is the maximal value of the plasticization force *F_plMAX_*. The value of this parameter has a direct influence on the power consumption of the main drive. However, the analysis performed leads to conclusions that the plasticization operation is most effective when the plasticization force in the second phase of the operation has a constant value (Figure 15). Due to this fact, a more detailed analysis of the plasticization characteristics is necessary. The analysis should result in the derivation of the operation parameters for which the plasticization process will be optimal.

### 4.4. Plasticization Force Modeling

To determine the effective parameters of the belt plasticization process, a mathematical model should be derived describing the dependence of the plasticization force *F_pl_* on basic technological parameters during plasticization (diameter *d*, plasticization velocity *v_pl_*, and the temperature of the hot plate *T_p_*) and displacement during plasticization *s*. This action can be performed on the basis of the obtained experimental results. The following ranges for the process of process parameters were adopted for the modeling:

- Diameter *d* which varies in a discrete way with values: 12 mm, 15 mm, and 18 mm;
- Temperature of the hot plate *T_p_* which varies continuously from 270 °C to 300 °C;
- Plasticization velocity *v_pl_*, which varies continuously from 4 mm/min to 10 mm/min.

**Figure 16:**
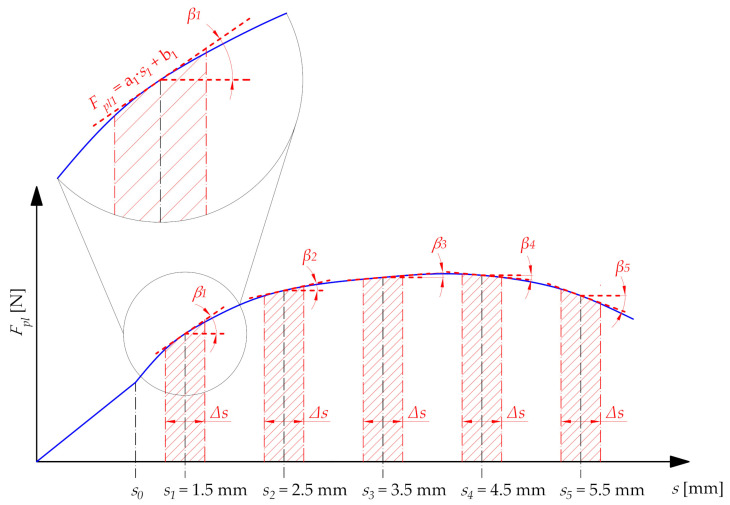
Exemplary real plasticization characteristic *F_pl_* (*s*) with the method of examination of nonsteady plasticization function: *F_pl_*_1_…*F_pl_*_5_—value of the plasticization force, *s*_0_—initial displacement (the border of the non-steady state), *s*_1_…*s*_5_—analysed control points, Δ*s*—considered range of displacement, *a*_1_…*a*_5_—directional coefficients of the approximation function, *b*_1_…*b*_5_—constants of the approximation functions, *β*_1_…*β*_5_—angle of inclination between the approximation function and plasticization characteristics, tangent at points *s*_1_…*s*_5_.

The determination of the mathematical model requires the analysis of the course of plasticization characteristics *F_pl_*(*s*), especially in the second phase (Figure 16). The most appropriate mathematical tool for this purpose is the examination of the monotonicity of the function. To do that, in the displacement domain, the five control points *s*_1_*…s*_5_ were determined. Their locations are determined by particular values of the plasticization distance *s*. At each control point, the tangent to the function *F_pl_*(*s*) was determined using linear regression. The functions obtained have the following form:

(3) $$F_{pl_{i}}=a_{i}\cdot s_{i}+b_{i}\;\mathrm{for}\;i=1\ldots 5$$

The coefficients of the straight line that is tangential to the initial function *F_pl_*(*s*) were determined using linear regression from the interval described by width Δ*s*:

(4) Δs=0.4 mm,

which is in the following correlation with control points:

(5) $$\mathrm{from}\;s_{i}-0.2\;\mathrm{mm}\;\mathrm{to}\;s_{i}+0.2\;\mathrm{mm}$$

in order to locate the middle of the interval precisely at point *s_i_*. Due to this, the assumptions about tangency in point *s_i_* are valid. The linear functions obtained have a determination coefficient *R*^2^ > 0.95 in relation to the initial function *F_pl_*(*s*) in the intervals analyzed. Therefore, it can be considered that these data are relatively well-suited, which mainly results from the relatively small width of the analyzed interval.

Therefore, with the accepted coordinates of the *s_i_* control points (1.5 mm; 2.5 mm; 3.5 mm; 4.5 mm; 5.5 mm), the mathematical model will be determined for the continuous interval from 1.5 mm to 5.5 mm—so as to obtain the best adjustment of the empirical data obtained in the form of a model.

**Figure 17:**
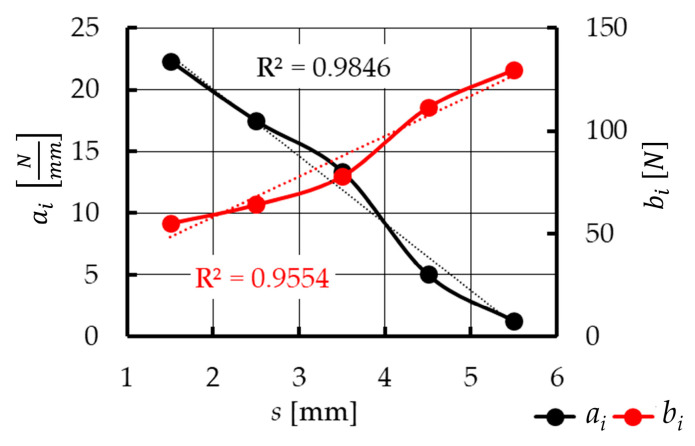
Exemplary correlation between the coefficients *a_i_* and *b_i_*, and the displacement *s* for the belt with diameter 12 mm, plasticized with velocity *v_pl_* = 10 mm/min and with the temperature of the hot plate *T_p_* = 285 °C.

Next, the dependence between coefficients of Equation (3) and the displacement *s* (Figure 17) was determined. These coefficients can be described using a linear function with a determination coefficient *R*^2^ > 0.95:

(6) $$a_{i}=a_{sa}\cdot s+b_{sa},$$

(7) $$b_{i}=a_{sb}\cdot s+b_{sb}.$$

On the basis there is the possibility to determine a function describing the correlation between the modeled plasticization force *F_pl_mod_* and the displacement *s*:

(8) $$F_{pl\_mod}=\left(a_{sa}\cdot s+b_{sa}\right)\cdot s+\left(a_{sb}\cdot s+b_{sb}\right)$$

where *a_sa_*, *b_sa_*, *a_sb_*, and *b_sb_* are coefficients that consider changes of the displacement or particular control points for a general model of the plasticization force.

**Figure 18:**
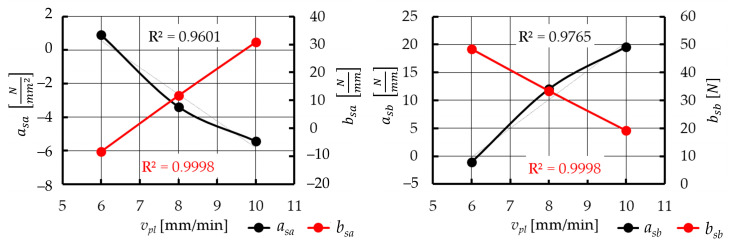
Exemplary correlation between the *a_sa_*, *b_sa_*, *a_sb_*, and *b_sb_* coefficients and the plasticization velocity *v_pl_* for the belt with 12 mm diameter, plasticized by the hot plate heated to temperature *T_p_* = 285 °C.

The Equation (8) obtained at this stage does not include any parameters of the plasticization operation (*d*, *T_p_*, *v_pl_*). Therefore, the correlation between the coefficients (*a_sa_*, *b_sa_*, *a_sb_*, and *b_sb_*) and the plasticization velocity *v_pl_* was determined (Figure 18) using a linear function with a determination coefficient *R*^2^ > 0.96:

(9) $$a_{sa}=a_{vasa}\cdot v_{pl}+b_{vasa}$$

(10) $$b_{sa}=a_{vbsa}\cdot v_{pl}+b_{vbsa}$$

(11) $$a_{sb}=a_{vasb}\cdot v_{pl}+b_{vasb}$$

(12) $$b_{sb}=a_{vbsb}\cdot v_{pl}+b_{vbsb}$$

On the basis of that, it is possible to determine a correlation between the modeled plasticization force *F_pl_mod_* and the displacement *s* and the plasticization velocity *v_pl_*:

(13) $$F_{pl\_mod}=\left[\left(a_{vasa}\cdot v_{pl}+b_{vasa}\right)\cdot s+\left(a_{vbsa}\cdot v_{pl}+b_{vbsa}\right)\right]\cdot s+\left(a_{vasb}\cdot v_{pl}+b_{vasb}\right)\cdot s+\left(a_{vbsb}\cdot v_{pl}+b_{vbsb}\right)$$

where *a_vasa_*, *b_vasa_*, *a_vbsa_*, *b_vbsa_*, *a_vasb_*, *b_vasb_*, *a_vbsb_*, and *b_vbsb_* are the coefficients that consider changes in the plasticization velocity *v_pl_* for previously determined coefficients of a general plasticization force model *a_sa_*, *b_sa_*, *a_sb_*, and *b_sb_* (Equation (8)).

**Figure 19:**
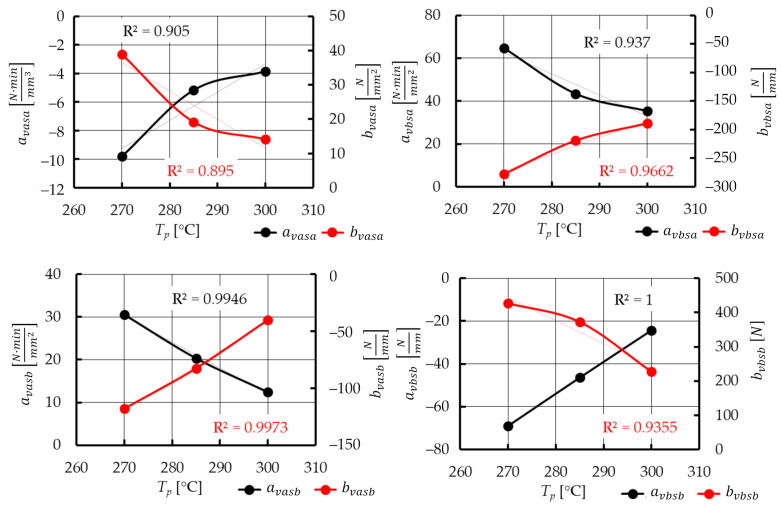
Exemplary correlation between the coefficients: *a_vasa_*, *b_vasa_*, *a_vbsa_*, *b_vbsa_*, *a_vasb_*, *b_vasb_*, *a_vbsb_*, and *b_vbsb_* and the temperature of the hot plate *T_p_* for the belt with diameter of 12 mm, plasticized with velocity *v_pl_* = 10 mm/min.

The resulting Equation (13) does not take into account the temperature of the hot plate *T_p_* as another continuous variable. To introduce this parameter into the plasticization force model, the dependencies between the coefficients (Equation (13)) and the temperature of the hot plate *T_p_* were derived (Figure 19). Using a linear function, with a determination coefficient *R*^2^ > 0.9, the coefficients of Equation (13) can be presented as:

(14) $$a_{vasa}=a_{tavasa}\cdot T_{p}+b_{tavasa}$$

(15) $$b_{vasa}=a_{tbvasa}\cdot T_{p}+b_{tbvasa}$$

(16) $$a_{vbsa}=a_{tavbsa}\cdot T_{p}+b_{tavbsa}$$

(17) $$b_{vbsa}=a_{tbvbsa}\cdot T_{p}+b_{tbvbsa}$$

(18) $$a_{vasb}=a_{tavasb}\cdot T_{p}+b_{tavasb}$$

(19) $$b_{vasb}=a_{tbvasb}\cdot T_{p}+b_{tbvasb}$$

(20) $$a_{vbsb}=a_{tavbsb}\cdot T_{p}+b_{tavbsb}$$

(21) $$b_{vbsb}=a_{tbvbsb}\cdot T_{p}+b_{tbvbsb}$$

As a consequence, the modeled plasticization force *F_pl_mod_* in relation to variable technological parameters (*T_p_* and *v_pl_*) and the displacement *s* can be calculated using the following function:

(22) $$F_{pl\_mod}=\{\left[\left(a_{tavasa}\cdot T_{p}+b_{tavasa}\right)\cdot v_{pl}+\left(a_{tbvasa}\cdot T_{p}+b_{tbvasa}\right)\right]\cdot s+\left(a_{tavbsa}\cdot T_{p}+b_{tavbsa}\right)\cdot v_{pl}+\;\left(a_{tbvbsa}\cdot T_{p}+b_{tbvbsa}\right)\}\cdot s+\{\left[\left(a_{tavasb}\cdot T_{p}+b_{tavasb}\right)\cdot v_{pl}+\left(a_{tbvasb}\cdot T_{p}+b_{tbvasb}\right)\right]\cdot s+\;\left(a_{tavbsb}\cdot T_{p}+b_{tavbsb}\right)\cdot v_{pl}+\left(a_{tbvbsb}\cdot T_{p}+b_{tbvbsb})\right\}$$

where: *a_tavasa_*, *b_tavasa_*, *a_tbvasa_*, *b_tbvasa_*, *a_tavbsa_*, *b_tavbsa_*, *a_tbvbsa_*, *b_tbvbsa_*, *a_tavasb_*, *b_tavasb_*, *a_tbvasb_*, *b_tbvasb_*, *a_tavbsb_*, *b_tavbsb_*, *a_tbvbsb_*, and *b_tbvbsb_* are the coefficients that consider changes in the hot plate temperature *T_p_* for previously determined coefficients of a general plasticization force model *a_vasa_*, *b_vasa_*, *a_vbsa_*, *b_vbsa_*, *a_vasb_*, *b_vasb_*, *a_vbsb_* and *b_vbsb_* (Equation (13)).

The function obtained 22 does not include the diameter of the belt *d*. This situation is intentional because the diameter of the belt *d* varies in a discrete way and depends on the current requirements. The aim of preparing this model does not concern optimization of the diameter *d* but refers to optimization of the process parameters for the required diameter *d*, which has a known value.

Based on the obtained model (Equation (22)), the modeled plasticization force *F_pli_mod_* was calculated. After that, a comparison was made between the modeled plasticization force *F_pil_mod_* and the experimental results *F_pli_exp_* for each of the control points *s_i_.* An exemplary list of the results, for a few control points, is presented in Table 2.

**Figure 20:**
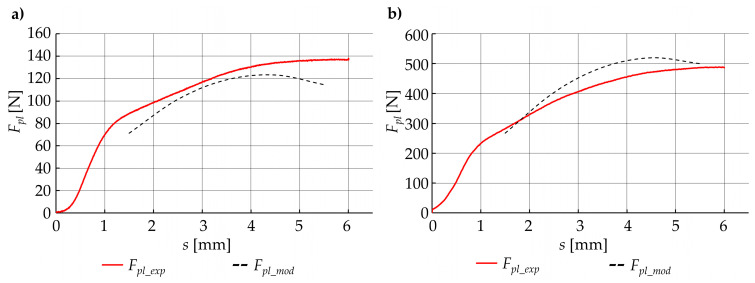
Examples of the characteristics of the plasticization force *F_pl_*(*s*) concerning the aspect of comparison between the values of empirical research (*F_pl_exp_*) and the derivative model (*F_pl_mod_*): (**a**) belt with diameter of 12 mm, hot plate temperature *T_p_* = 285 °C, plasticization velocity *v_pl_* = 10 mm/min, (**b**) belt with diameter of 18 mm, hot plate temperature *T_p_* = 285 °C, plasticization velocity *v_pl_* = 8 mm/min.

A graphical comparison of the developed model with empirical data (Figure 20) was also made. Based on the comparative analysis of the results of the developed model and the results of empirical tests (Table 2 and Figure 20), it can be seen that the results of the developed model deviate from the results obtained from empirical research. The absolute difference *δF_mod_* between the modeled force *F_pl_mod_* and those that were collected from the experiment *F_pl_exp_* were calculated from the following formula:

(23) $$\delta F_{mod}=\frac{\left|F_{pl\_exp}-F_{pl\_mod}\right|}{F_{pl\_exp}}\cdot 100\%\;$$

and in some cases, exceeded 20%. Based on the graphical analysis (Figure 20), it can be seen that the deviations obtained mainly resulted from a different shape of the modeled plasticization force *F_pl_mod_* relative to the experimental results *F_pl_exp_*. This proves that the adjustment of this function is not effective in the entire displacement range. Although the coarse force values agree, the total reproduction of the course is not accurate enough.

**Figure 21:**
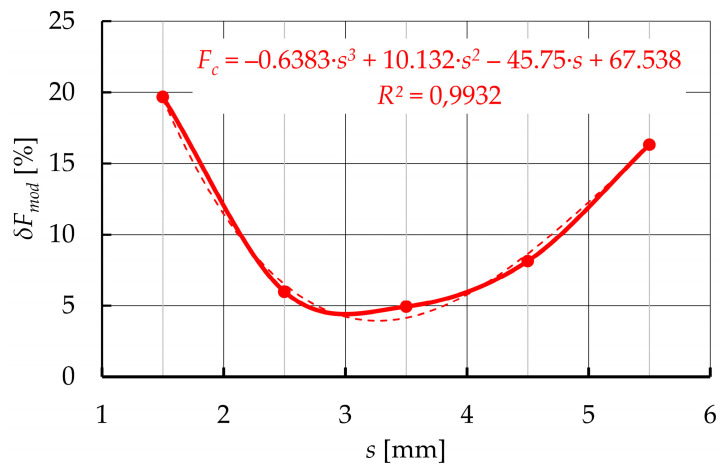
Exemplary correction function that describes the absolute error between the experimental plasticization force *F_pl_exp_* and calculated model *F_pl_mod_* for the belt with diameter of 12 mm, plasticized with velocity *v_pl_* = 10 mm/min, and with the hot plate temperature *T_p_* = 285 °C.

To obtain a better fit of the model, the correction function *F_c_*(*s*) has been developed for each of the 27 groups of parameters. Using the percentage deviations of the model from the experimental data Equation (23), and the non-linear regression method, it is possible to derive the error function, which can be described by a 3-degree polynomial, with the determination coefficient *R*^2^ > 0.99 (Figure 21). The developed correction function can be presented as:

(24) $$F_{c}=a_{c}\cdot s^{3}+b_{c}\cdot s^{2}+c_{c}\cdot s+d_{c}\;$$

Considering the fact that the correction function was derived on the basis of the percentage error calculation (Equation (23)), the corrected function *F_pl_mod_c_*, which describes the plasticization force from the corrected model, can be presented as:

(25) $$F_{pl\_mod\_c}=F_{pl\_mod}\cdot \left(\frac{100+F_{c}}{100}\right)$$

After the calculations of the corrected model, the absolute difference *δF_mod_c_* between the corrected model force *F_pl_mod_c_* and the ones collected from the experiment *F_pl_exp_* can be calculated in the same way as previously Equation (23).

**Figure 22:**
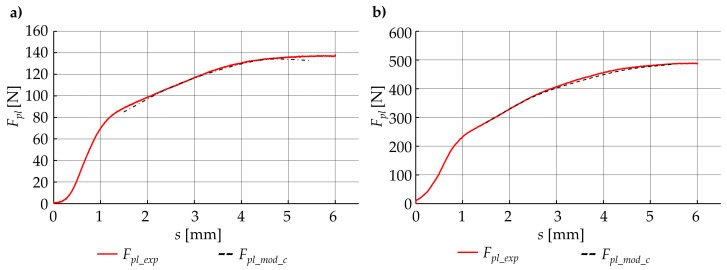
Examples of the characteristics of the plasticization force *F_pl_*(*s*) concerning the aspect of comparison between values of experimental research (*F_pl_exp_*) and from the derivative model (*F_pl_mod_*): (**a**) belt with diameter of 12 mm, hot plate temperature *T_p_* = 285 °C, plasticization velocity *v_pl_* = 10 mm/min, (**b**) belt with diameter of 18 mm, hot plate temperature *T_p_* = 285 °C, plasticization velocity *v_pl_* = 8 mm/min.

Comparison of experimental results with data obtained from the adjusted model (Table 2 and Figure 22) shows that the use of the correction function allows better fitting of the developed model to empirical parameters. Due to this fact, for all the cases of a set of technological parameters, the corrected model error did not exceed 5%.

However, it should be noted that both the *F_pl_mod_* function and the correction function *F_c_*, and, consequently, the corrected model *F_pl_mod_c_*, are valid for the assumed range of plasticization parameters. This is the consequence of developing a model on the basis of experimental results. Notwithstanding, it should be noted that for the purpose of obtaining an effectively designed drive system, this approach is enough.

### 4.5. Optimization of the Technological Parameters

With a designated function describing the dependence between the plasticization force *F_pl_* and basic technological parameters during plasticization (plasticization velocity *v_pl_*, temperature of the hot plate *T_p_*, and displacement during plasticization *s*), it is possible to optimize the technological parameters of this operation during the second phase (Figure 15) in order to obtain the desired course of this operation. From the design point of view, the most advantageous situation is to obtain the maximum energy efficiency. Considering this fact and on the basis of previously drawn conclusions, it is possible to specify the following target functions:

- Minimizing the plasticization force *F_pl_* during the entire operation. This target function can be described in a mathematical way as:

(26) $$\min F_{pl}$$

To realize this condition in a practical way, it is necessary to check the value of the maximum value of the plasticization force at each control point:

(27) $$\max F_{pli}\;\mathrm{for}\;i=1\ldots 5$$

and compare it with results for different technological parameters (*v_pl_* and *T_p_*). This operation makes it possible to check for which parameters the force value will be the smallest Equation (26).

Based on the results obtained (Figure 14 and Figure 15), it can be assumed that the maximal plasticization force *F_pl_max_* is present during the whole plasticization process during the second phase (at the beginning, for the small velocity *v_pl_* or the higher plate temperature *T_p_*—Figure 14a; somewhere in the middle, for the intermediate velocity *v_pl_* or the intermediate plate temperature *T_p_*—Figure 14b; at the end, for the higher velocity *v_pl_* or the smaller plate temperature *T_p_*—Figure 14c).

- Constant value of the plasticization force *F_pl_*(*s*) is possible, in the whole calculation range:

(28) $$F_{pl}\left(s\right)≅const\;$$

At this point, the assumption that the maximal percentage difference between the plasticization force values at each control point should not exceed 10% have to be made. Considering the introduction of control points (discretization) at the second phase of plasticization characteristics (Figure 16), this target function can be mathematically formulated as:

(29) $$\delta F_{pli}\left(s\right)=\frac{\left|F_{mod\_c\_i}-F_{mod\_c\_j}\right|}{F_{mod\_c\_i}}\cdot 100\%<10\%,$$

and in the further considerations can be called *control function*.

**Figure 23:**
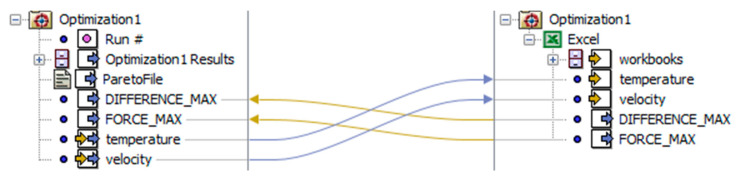
Exemplary view of the connection between particular parameters during optimization using MS Excel and Simulia Isight software.

To perform the optimization, with the specified target functions, the connection was made between the MS Excel software and the Similia Isight environment (Figure 23). The spreadsheet was responsible for performing the calculations of the values of the functions *F_pl_*(*s*) and *δF_pl_*(*s*) for five control points *s_i_*, using standard calculation procedures. The Simulia Isight environment, with a built-in optimization module [60], had three basic functions:

- Sequential substitution of the technological parameters of the plasticization operation (*v_pl_*, *T_p_*, and *s*) to call for further iterations of calculations in MS Excel software;
- Collecting the results in the form of values of the current plasticization force *F_pl_* and the current value of the control function *δF_pl_*;
- Optimization of the technological parameters (*v_pl_* and *T_p_*) to fulfill the target functions (27)–(29).

Optimization was performed using the NCGA genetic algorithm (Neibourhood Cultivation Genetic Algorithm). The following parameters were adopted:

- Population of 50 individuals;
- 200 generations;
- Double-crossing allowed;
- Scaling factor (due to expected difference in a range of obtained quantities):

(30) $$SFF_{pl}=100$$

(31) $$SF\delta F_{pli}=10$$

These assumptions resulted in the calculation of 10,000 iterations.

**Figure 24:**
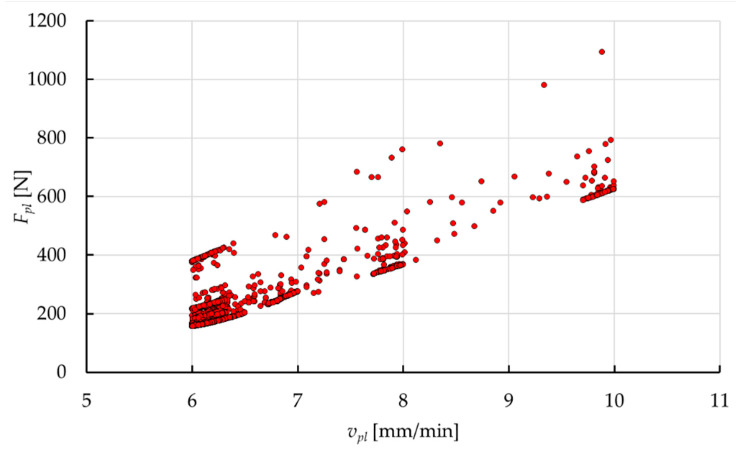
Exemplary map of the calculation points during the plasticization force calculations using the genetic algorithm for the belt with diameter of 18 mm and different plasticization velocity *v_pl_*.

Figure 24 shows an example map of computing points resulting from the operation of the genetic algorithm. According to the developed model, the dependence of force during plasticization *F_pl_* is non-linear in relation to the plasticization velocity *v_pl_*, which has found a mapping in the general trend of the arrangement of the computing points. According to the distribution of the calculated points presented, the algorithm focused on the places where there is the smallest value of the plasticization force *F_pl_*. This is an expected effect of the work of the genetic algorithm during optimization, considering the assumed target functions.

**Figure 25:**
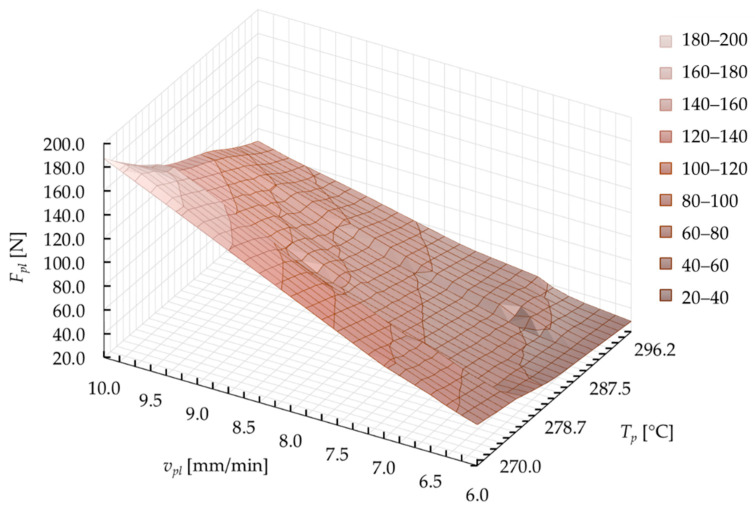
Exemplary results in the form of the plasticization force surface *F_pl_* in correlation with the hot plate temperature *T_p_* and the plasticization velocity *v_pl_* for the belt with diameter of 18 mm.

Figure 25 presents an exemplary surface that is formed by values of the plasticization force *F_pl_*. The results are presented in correlation with the technological parameters (*v_pl_* and *T_p_*). This map was obtained on the basis of the model calculations during optimization at individual computing points. The increase in the value of the plasticization force *F_pl_* with increasing plasticization velocity *v_pl_* and the reduction of the hot plate temperature *T_p_* gives an expected effect, which was also confirmed during the experiment (Figure 15).

The correlation obtained shows that there was an almost complete coverage of the range in variability of the parameters during calculations. Unfortunately, this type of graph does not show the diversified density of individual computing points (Figure 24). This feature is natural for calculations using a genetic algorithm, due to the densification of the computing points in the area around the optimal solution, with keeping the restrictions (allowed values of the parameters).

**Figure 26:**
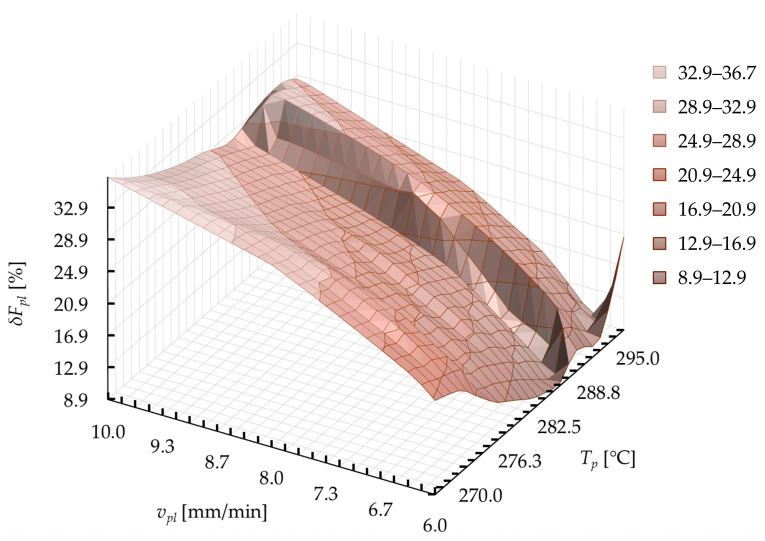
Exemplary results in the form of the surface of the function *δF_pli_* in correlation with the hot plate temperature *T_p_* and the plasticization velocity *v_pl_* for the belt with diameter of 18 mm.

Figure 26 shows the exemplary surface formed by the values of the control function *δF_pli_* in correlation with the plasticization parameters (*v_pl_* and *T_p_*). The map is prepared on the basis of the conversion of the developed model at individual computing points during optimization. The middle area of this surface, in which the decrease in the function is observed, is the place where the solutions of the second target function should be found. In this area, the smallest value of the difference in the plasticization force between subsequent control points is present.

The final results of the optimization are shown in Table 3. On the basis of optimization with a genetic algorithm, the most effective parameters of the plasticization operation (*v_pl_eff_* and *T_p_eff_*) can be derived. They ensure the lowest possible value of the plasticization force *F_pl_eff_* and relatively constant flow of the plasticization characteristics.

On the basis of the obtained parameters, the value of the effective plasticization force from the prepared model (*F_pli_eff_mod_c_*) and maximal difference between the control points from the model (*δF_pli_eff_mod_c_*) can be obtained (using these values optimization was conducted). To verify the model used, the experimental plasticization procedure (Figure 12 and Figure 13) was repeated for effective parameters (*v_pl_eff_* and *T_p_eff_*). On the basis of this experiment, the maximal value of effective plasticization force (*F_pli_eff_exp_*) and maximal difference between control points (*δF_pli_eff_exp_*) were derived. Consequently, to verify the model, the deviation from the model (*δF_eff_mod_c_*) was determined. The value of these parameters does not exceed 5%, so it can be assumed that the model is precise enough.

According to the results obtained, in the case of comparing different diameters *d* of the plasticized belt, a higher effect is obtained by changing the temperature of the hot plate *T_p_* than by changing the plasticization velocity *v_pl_*.

## 5. Estimation of Power Consumption

### 5.1. Component Selection

To carry out the design process properly, it is necessary to make the required engineering calculations. Unfortunately, before starting them, the most typical situation is that the demanded values are not precisely defined. In this case, there is a necessity to prepare an initial draft of designed mechanisms, without details connected with, for example, strength aspects. Consequently, the designer should make some assumptions related with some estimated geometrical dimensions or sizing of particular components.

For the automated hot plate welder, the authors prepared an initial 3D model of the whole construction on the basis of previous experience. Accordingly, the following types of components were assumed:

- BLDC electric motor (DB series) with built-in planetary gear (GP series) from Nanotec [61];
- Jaw coupling with elastomeric insert (Rotex series) from KTR [62];
- Ball screw mechanisms—a screw with a nut (FSC type with GFD housing) from Hiwin [63]. The initial parameters of the ball screw were set on the basis of initial buckling calculations. The screw to drive the movable belt holder has an effective diameter *d*_1_ = 16 mm and a pitch *p*_1_ = 10 mm. The screw to drive the heating unit has the same effective diameter *d*_2_ = *d*_1_ and pitch *p*_2_ = 5 mm. Due to that, the assumed correlation Equation (1) between the values of the displacement obtained during rotary movement is obtained;
- Angular bearings with housings (SFA type) from Hiwin [63];
- Radial bearing with housings (SLA type) from Hiwin [63];
- Linear guideways with balls as rolling elements (MSA type) from PMI [64].

After preparing the initial 3D model, the geometrical parameters were measured. Their values are presented in Table 4. On the basis of the model, the masses of particular units can be determined:

- Approximate mass of the movable belt holder is *m_mbh_* = 22 kg;
- Approximate mass of heating unit is *m_hu_* = 11 kg.

Based on the available content from the manufacturers [61,62,63,64], the following values of efficiency and energy losses of particular units can be assumed:

- Planetary gear is *η_pg_* = 0.95;
- Jaw coupling is *η_jc_* = 0.98;
- Angular bearing is *η_ab_* = 0.99;
- Radial bearing is *η_rb_* = 0.99;
- Screw mechanism with pitch is *p*_1_—friction coefficient *η_sm_*_1_ = 0.92;
- Screw mechanism with pitch is *p*_2_—friction coefficient *η_sm_*_2_ = 0.85;
- Friction coefficient in linear guideways is *μ*_1_ = 0.005.

The assumptions connected with these parameters will make it possible to perform the engineering calculations required to estimate the power consumption of the main drive unit. Additionally, for this purpose, it is necessary to consider the dynamical behaviour of key components to assume the proper state of the load.

### 5.2. Estimation of the Power Consumption

The power consumption of the main drive of the automated hot plated welder should be understood as a value of the required power of the main electric motor, which is responsible for driving the movable belt holder and heating unit. In general, calculating the power requirement for the drive system of the technological device can be carried out in many ways. In many cases, computational methods related to motion dynamics analysis are used, particularly the Lagrange equations of the second type [65,66,67]. This is due to the large dynamics of the operation of some mechanisms, which is natural for such solutions as:

- Mechanisms of technological devices that work with high rotational or linear speed, especially when working speed must be achieved in a short time from the start-up [65];
- Mobile devices capable of overcoming various types of obstacles and moving with relatively high velocity, while achieving relatively high acceleration values [66];
- Positioning and stabilization mechanisms, especially with PID regulation [67].

The driving system of the automated welding machine seems to be a little different. The achieved velocities of particular components (e.g., movable belt holder) are relatively small (row of several millimeters per minute). In addition, in this case, there are no relatively restrictive requirements related to accelerating and braking time in the initial and final phases of motion.

**Figure 27:**
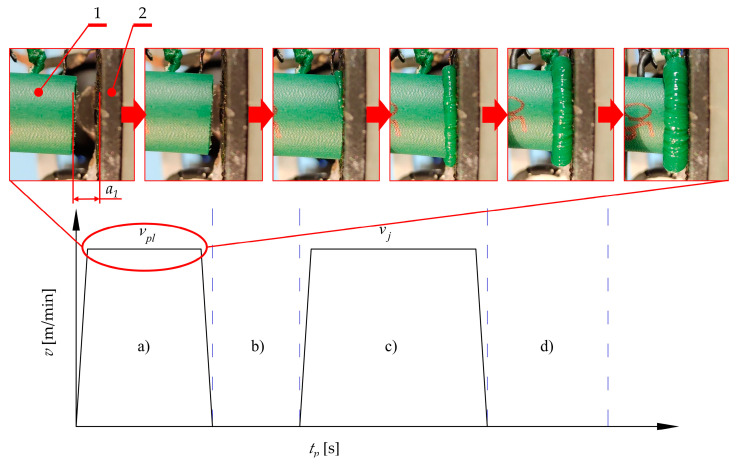
The cyclogram of the hot plate welding process with illustration of real plasticization process: (a) plasticization, (b) hot plate removal, (c) pressing operation, (d) cooling; *v*—velocity, *v_pl_*—plasticization velocity, *v_j_*—joining velocity, *t_p_*—process time, *a*_1_—initial distance between the end of the belt and the hot plate; 1—belt, 2—hot plate.

Based on the developed welding method (Figure 2), the course of this operation can be described using a cyclogram (Figure 27), taking into account the correlation between velocity *v* (*v_pl_* or *v_j_*), achieved during the welding operation, and process time *t_p_*.

In the considered range of the welding operation, during the plasticization of the material (a), the plasticization velocity *v_pl_* is constant over a wide range of process time *t_p_* intended for this operation. Small deviations from this situation are caused by the accelerating and braking of the movement of the mechanisms, at the initial and final section of motion. The overall time of these phenomena is estimated at no more than 10% of the duration of the entire plasticizing operation. During the uniform motion (with constant plasticization velocity *v_pl_*), the initial gap *a*_1_, which is present between the belt (1) and the hot plate (2), is cleared. Consequently, the surfaces of these elements (1 and 2) come into contact and the proper plasticization operation starts. According to observations made during experiments, the moment of entry into contact between the belt and the hot plate is the time when the largest increase in technological force occurs (phase I in Figure 15). Therefore, it is possible to adopt a simplification in which plasticization of the material starts at the time of a monotonous, uniform motion of the movable belt holder and the heating unit (10 and 11 in Figure 6). According to that, the calculations of the load which acts on individual components of the drive system, for determining the value of the power consumption of particular units, can be performed using the static load analysis.

Due to the three-dimensional state of the load distribution and spatial arrangements of the support points, both for the movable belt holder and the heating unit, the analysis of the distribution of forces to determine the load of the guideways and calculate the drive forces *F_d_*_1_ and *F_d_*_2_ (Figure 8) is not a trivial issue. It requires analyzing the impact of the moments of the forces that affect the guideways. The complexity of the issue particularly applies to the heating unit in which there is a statically indeterminate system of force, due to the presence of three supports. Therefore, the analysis of these systems requires the determination of force components and moments in individual planes, which makes it possible to determine, for example, forces that load the guideways.

After completing the necessary calculations for the movable belt holder, it can be concluded that the maximum force that acts for its linear guiding is present in the right carriage (Figure 10). Its value can be calculated from the dependence:

(32) $$F_{g\_mbh\_MAX}=\frac{m_{mbh}}{w_{1}}\cdot g\cdot \left(w_{1}-w_{4}\right)+\left[\left(F_{pl}+m_{mbh}\cdot g\cdot \mu_{1}\right)\cdot h_{1}+m_{mbh}\cdot g\cdot \left(\frac{1}{2}\cdot l_{4}+l_{2}\right)-F_{pl}\cdot h_{4}\right]\cdot \frac{1}{l_{4}}$$

In the case of a 3-trolley leading a heating unit, the force with the highest value affects the right bottom carriage (Figure 10). Its value can be calculated from the dependence:

(33) $$F_{g\_hu\_MAX}=m_{hu}\cdot g+\frac{F_{pl}\cdot h_{5}+m_{hu}\cdot g\cdot l_{6}-\left[\left(F_{pl}+m_{hu}\cdot g\cdot \mu_{1}\right)\cdot \left(h_{4}+h_{5}-h_{6}\right)\right]}{h_{4}+h_{5}}+m_{hu}\cdot g\cdot \frac{w_{5}}{w_{2}+w_{1}}$$

Designated values make it possible to perform the final selection of the size of the guide. To calculate the values of the drive forces (*F_d_*_1_ and *F_d_*_2_), it must be taken into account that all forces that load guideways cause friction resistance, which must be overcome by the drive system. After considering these facts, and the resulting possibility of simplifying the calculations, the values of the force needed to drive the movable belt holder *F_d_*_1_ and to drive the heating unit *F_d_*_2_ can be calculated using the following dependencies:

(34) $$F_{d1}=F_{pl}+m_{mbh}\cdot g\cdot \mu_{1}$$

(35) $$F_{d2}=F_{pl}+m_{hu}\cdot g\cdot \mu_{1}$$

After determining the value of these forces, the estimation of the power consumption of the drive system can start. To do this correctly, the transformation of energy in the screw mechanism (from linear to rotary motion) and the efficiency of individual structural nodes should be taken into account (Figure 8). Considering the correlations between operational velocities:

(36) $$v_{1}=2\cdot v_{pl}$$

(37) $$v_{2}=v_{pl}$$

the power balance for the screw mechanism of the movable belt holder takes the following form:

(38) $$P_{fs}\cdot \eta_{sm1}=P_{d1}=F_{d1}\cdot v_{1}=F_{d1}\cdot 2\cdot v_{pl}$$

For the heating unit, the following correlation should be used:

(39) $$P_{ss}\cdot \eta_{sm2}=P_{d2}=F_{d2}\cdot v_{2}=F_{d2}\cdot v_{pl}$$

Considering the energy losses of particular parts (bearings, couplings) and mechanisms (planetary gear), the overall output power of the electric motor can be calculated from the following correlation:

(40) $$P_{em}=\frac{1}{\eta_{pg}\cdot \eta_{jc}\cdot \eta_{ab}\cdot \eta_{rb}}\cdot \left(P_{fs}+\frac{P_{ss}}{\eta_{jc}\cdot \eta_{ab}\cdot \eta_{rb}}\right)$$

According to the previous dependencies (38) and (39), the power consumption takes the following form:

(41) $$P_{em}=\frac{1}{\eta_{pg}\cdot \eta_{jc}\cdot \eta_{ab}\cdot \eta_{rb}}\cdot \left(\frac{F_{d1}\cdot 2\cdot v_{pl}}{\eta_{sm1}}+\frac{F_{d2}\cdot v_{pl}}{\eta_{jc}\cdot \eta_{ab}\cdot \eta_{rb}\cdot \eta_{sm2}}\right)$$

and, finally, using the technological parameters and properties of the initial design, leads to the final form of the dependency for the power consumption:

(42) $$P_{em}=\frac{1}{\eta_{pg}\cdot \eta_{jc}\cdot \eta_{ab}\cdot \eta_{rb}}\cdot \left[\frac{\left(F_{pl}+m_{mbh}\cdot g\cdot \mu_{1}\right)\cdot 2\cdot v_{pl}}{\eta_{sm1}}+\frac{\left(F_{pl}+m_{hu}\cdot g\cdot \mu_{1}\right)\cdot v_{pl}}{\eta_{jc}\cdot \eta_{ab}\cdot \eta_{rb}\cdot \eta_{sm2}}\right]$$

The screw mechanism makes it possible to transform the energy from rotational movement into translational. The required rotational speed of the screws *n_s_* (Figure 8) can be calculated from the following formula:

(43) $$n_{s}=\frac{2\cdot v_{pl}}{p_{1}}=\frac{v_{pl}}{p_{2}}$$

Performed calculations will allow one to make an effective choice of the electric motor, concerning the aspect of the required power and rotational speed and, consequently, the gear ratio of the planetary gear. The results of the calculations for the optimized parameters are presented in Table 5 (plasticization force for this purpose was determined from the derivate model—*F_pl_eff_mod_c_*, for simplification in Table 5 it is designated as for plasticization force *F_pl_*). Obtained results were expected. Increase in plasticization force leads to the increase in power consumption.

## 6. Conclusions

The research and design work carried out was aimed at developing and constructing the drive system of an automated hot plate welder. Using the connection between the following research and development tools:

- Conceptual design works;
- Experimental research, which is used for mapping a technological process to determine technological parameters;
- Mathematical modeling of the course of technological operation, in order to determine the load forces;
- Optimization in terms of energy efficiency process, using a genetic algorithm;
- Analysis of the construction of the structure to determine the forces necessary for the driving of individual components;
- The final calculation of power consumption, a structural problem was solved, which consisted of determining the power of the drive components. In addition, as a result of the work carried out, a relatively simple structural solution of the device was obtained, especially concerning the aspect of the drive system. The modularity of the structure allows the device to expand with, among others, the unit for butt removal. The works made are a fragment of a wider spectrum of considerations that have been committed on the occasion of designing this device.

Experimental studies of plasticization of the material on the hot plate resulted in the dependence between the plasticization force *F_pl_* and the displacement *s*. On their basis, it was possible to determine the behavior of the material in the welding process. Particularly interesting is the fact that it is possible to maintain a constant value of the plasticization force *F_pl_* in the second phase of the process. Due to this fact, there is the possibility to choose technological parameters so that the welding process using the designed device will be maximally effective. The main goal is to obtain a proper degree of material plasticization without an adverse effect of the flowing material from the workspace.

The mathematical model of the belt plasticization force *F_pl_*, depending on the displacement *s*, developed on the basis of experimental results, has a certain error due to some imperfections of the method (approximating the results using the regression method). Using the correction function *F_c_*, developed on the percentage of the model deviation from the original results, causes the difference between the model and the experimental results to be sufficiently small, for the needs of the drive system design.

In the authors’ opinion, the genetic algorithm worked well in the optimization. The density of computing points in the area of optimal solutions caused the expected solutions found.

In the following steps, optimization tasks related to the design of this structure can be extended to the issue related to energy consumption, especially research on the optimization of technological parameters with regards to the aspect of the heating of the hot plate.

## Figures and Tables

**Table 1:** The empirical research parameters, for analysed 27 measuring points.

Parameter	Designation	Value								
Belt diameter	*d* [mm]	12			15			18		
Hot plate temperature	*T_p_* [°C]	270 ± 1	285 ± 1	300 ± 1	270 ± 1	285 ± 1	300 ± 1	270 ± 1	285 ± 1	300 ± 1
Plasticization velocity	*v_pl_* [mm/min]	6, 8, 10	6, 8, 10	6, 8, 10	4, 6, 8	6, 8, 10	6, 8, 10	4, 6, 8	6, 8, 10	6, 8, 10
Specimen height	*h* [mm]	25 ± 0.1								
Specimen protrusion distance	*h*_1_ [mm]	10 ± 0.1								
Maximal displacement	*s_MAX_* [mm]	6 ± 0.01								

**Table 2:** The results of the comparison between the initial modeled plasticization force *F_pl_mod_* and experimental results *F_pl_exp_*, and corrected model of plasticization force *F_pl_mod_c_* for a few control points *s_i_*.

Parameter	Designation	Value								
Belt diameter	*d* [mm]	12			15			18		
Hot plate temperature	*T_p_* [°C]	270 ± 1	285 ± 1	300 ± 1	270 ± 1	285 ± 1	300 ± 1	270 ± 1	285 ± 1	300 ± 1
Plasticization velocity	*v_pl_* [mm/min]	6	8	10	8	10	6	6	10	8
Location of measurement point	*s* [mm]	2.5	3.5	4.5	2.5	1.5	3.5	2.5	4.5	1.5
Force from experimental research	*F_pli_exp_* [N]	56.918	72.520	88.886	253.747	201.968	86.973	312.758	741.677	246.999
Force from modeling	*F_pli_mod_* [N]	45.193	73.199	85.927	300.144	210.657	81.419	316.090	784.093	215.600
Deviation of standard model	*δF_mod_* [%]	**20.60**	**0.94**	**3.33**	**18.28**	**4.30**	**6.39**	**1.07**	**5.72**	**12.71**
Force from corrected model	*F_pli_mod_c_*	54.409	72.111	89.847	246.347	201.192	86.175	313.959	744.032	243.062
Deviation of corrected model	*δF_mod_c_* [%]	**4.41**	**0.56**	**1.08**	**2.92**	**0.38**	**0.92**	**0.38**	**0.32**	**1.59**

**Table 3:** The results of the NCGA optimization for three analyzed diameters of the belt.

Parameter	Designation	Value		
Belt diameter	*d* [mm]	12	15	18
Effective hot plate temperature	*T_p_eff_* [°C]	**277.5**	**292.5**	**300**
Effective plasticization velocity	*v_pl_eff_* [mm/min]	**6.45**	**6.49**	**6.29**
Effective plasticization force from corrected model	*F_pli_eff_mod_c_* [N]	56.90	107.55	179.86
Maximal difference between control points from model	*δF_pli_eff_mod_c_* [%]	8.24	6.92	8.35
Effective plasticization force from experiment	*F_pli_eff _exp_* [N]	58.58	103.32	185.56
Maximal difference between control points from experiment	*δF_pli_eff_exp_* [%]	7.95	8.03	8.47
Deviation of corrected model	*δF_eff_mod_c_* [%]	2.87	3.93	3.25

**Table 4:** The dimensions of the movable belt holder and heating unit assumed for calculations (Figure 9, Figure 10 and Figure 11).

Dimension	Value [mm]	Dimension	Value [mm]	Dimension	Value [mm]
*h*_1_	26	*l*_1_	155	*w*_1_	220
*h*_2_	115	*l*_2_	8	*w*_2_	50
*h*_3_	255	*l*_3_	158	*w*_3_	295
*h*_4_	240	*l*_4_	72	*w*_4_	70
*h*_5_	160	*l*_5_	40	*w*_5_	133
*h*_6_	33	*l*_6_	3	-	-

**Table 5:** The results of the power consumption calculations.

Parameter	Designation	Value		
Belt diameter	*d* [mm]	12	15	18
Effective hot plate temperature	*T_p_* [°C]	**277.5**	**292.5**	**300**
Effective plasticization velocity	*v_pl_* [mm/min]	**6.45**	**6.49**	**6.29**
Plasticization force	*F_pl_* [N]	56.90	107.55	179.86
Force to drive movable belt holder	*F_d_*_1_ [N]	57.98	108.63	180.94
Force to drive heating unit	*F_d_*_2_ [N]	57.44	108.09	180.40
Required rotational speed of the screw	*n_s_* [rpm]	1.290	1.298	1.258
Overall power consumption (required power of the electric motor)	*P_em_* [W]	0.0231	0.0437	0.0706

## Data Availability

Not applicable.
